# Comparison of biologics and small-molecule drugs in axial spondyloarthritis: a systematic review and network meta-analysis

**DOI:** 10.3389/fphar.2023.1226528

**Published:** 2023-10-24

**Authors:** Erye Zhou, Jian Wu, Keqin Zeng, Mingjun Wang, Yufeng Yin

**Affiliations:** Department of Rheumatology, The First Affiliated Hospital of Soochow University, Suzhou, Jiangsu, China

**Keywords:** biologics, small-molecule drugs, axial spondyloarthritis, systematic review, network meta-analysis

## Abstract

**Background:** Biologics and small-molecule drugs have become increasingly accepted worldwide in the treatment of axial spondyloarthritis (axSpA), including ankylosing spondylitis (AS) and non-radiographic axial spondyloarthritis (nr-axSpA). However, a quantitative multiple comparison of their efficacy and safety is lacking. This study aims to provide an integrated assessment of the relative benefits and safety profiles of these drugs in axSpA treatment.

**Methods:** We included randomized clinical trials that compared biologics and small-molecule drugs in the treatment of axSpA patients. The primary outcomes assessed were efficacy, including the Assessment of SpondyloArthritis International Society (ASAS) improvement of 20% (ASAS20) and 40% (ASAS40). Safety outcomes included treatment-emergent adverse events (TEAEs) and serious adverse events (SAEs). We used the surface under the cumulative ranking (SUCRA) curve value and ranking plot to evaluate and rank clinical outcomes and safety profiles of different treatments. The two-dimensional graphs were illustrated to visually assess both the efficacy (horizontal axis) and safety (vertical axis) of each intervention.

**Results:** Our analysis included 57 randomized clinical trials involving a total of 11,787 axSpA patients. We found that seven drugs (TNFRFc, TNFmAb, IL17Ai, IL17A/Fi, IL17RAi, JAK1/3i, and JAK1i) were significantly more effective in achieving ASAS20 response compared to the placebo (PLA). Except for IL17RAi, these drugs were also associated with higher ASAS40 responses. TNFmAb demonstrated the highest clinical response efficacy among all the drugs. Subgroup analyses for AS and nr-axSpA patients yielded similar results. IL17A/Fi emerged as a promising choice, effectively balancing efficacy and safety, as indicated by its position in the upper right corner of the two-dimensional graphs.

**Conclusion:** Our findings highlight TNFmAb as the most effective biologic across all evaluated efficacy outcomes in this network meta-analysis. Meanwhile, IL17A/Fi stands out for its lower risk and superior performance in achieving a balance between efficacy and safety in the treatment of axSpA patients.

## 1 Introduction

Axial spondyloarthritis (axSpA), characterized by inflammatory back pain and stiffness, is one of the most prevalent rheumatic conditions (Danve and Deodhar, 2022). AxSpA includes radiographic axSpA, commonly known as ankylosing spondylitis (AS), and non-radiographic axSpA (nr-axSpA) (Sieper and Poddubnyy, 2017). Current guidelines recommend non-pharmacological therapies as the primary approach to managing axSpA, alongside pharmacological treatments such as non-steroidal anti-inflammatory drugs (NSAIDs) or conventional synthetic disease-modifying anti-rheumatic drugs (csDMARDs) (Ramiro et al., 2022). Although these interventions may offer palliation of signs and symptoms, they have shown limited efficacy in reducing radiographic damage and modifying disease progression (Danve and Deodhar, 2022).

The development of targeted biologic therapies, including biologics, such as TNF-α inhibitors and IL-17 inhibitors, and small-molecule drugs, primarily JAK inhibitors, has revolutionized the clinical management of axSpA (Sunzini et al., 2022; Webers et al., 2022; Caso et al., 2023). Recent clinical trials and pairwise meta-analyses have demonstrated that these drugs offer significant clinical benefits to patients by promptly suppressing inflammation and targeting molecules that stimulate bone formation (Sieper and Poddubnyy, 2017; Yin et al., 2020; Lawson et al., 2021; Li et al., 2022). However, it is worth noting that, to date, there has been a notable lack of comprehensive head-to-head comparisons between these drugs (Giardina et al., 2010; van der Heijde et al., 2018b). This limitation leaves clinicians with a multitude of options to consider when prescribing pharmacotherapy (Cantini et al., 2017).

To bridge this gap, network meta-analysis is often employed to support evidence-based decision-making (Li et al., 2011). Network meta-analysis extends the principles of pairwise meta-analysis to evaluate multiple treatments by combining both direct and indirect comparisons across trials that share a common comparator, such as placebo (PLA) (Li et al., 2011). Several network meta-analyses have already been conducted to assess the performance of biologics and small-molecule drugs in axSpA (Betts et al., 2016; Deodhar et al., 2020a; Cao et al., 2022; Lee, 2022). However, more recent clinical trials have introduced additional drugs, including brodalumab (an IL-17 receptor A antibody, IL17RAi) (Wei et al., 2021a), upadacitinib (a JAK1-specific inhibitor, JAK1i) (Deodhar et al., 2022), and apremilast (a phosphodiesterase 4 inhibitor, PDE4i) (Taylor et al., 2021). Moreover, there exists a dearth of comparative efficacy studies for these drugs in the management of nr-axSpA.

Our study aimed to comprehensively evaluate the efficacy and safety of biologics and small-molecule drugs in axSpA patients, including both AS and nr-axSpA, by analyzing data from randomized clinical trials with placebo or active controls.

## 2 Methods

### 2.1 Registration and ethics

This study was designed and performed based on the methods and recommendations from the Preferred Reporting Items for Systematic Reviews and Meta-analyses for Network Meta-analysis (PRISMA-NMA) reporting guidelines (Hutton et al., 2015). The study protocol has been drafted *a priori* and registered in PROSPERO (CRD42022378343). We declare that all included data are available within the article and Supplementary Material.

### 2.2 Search strategy

The eligible studies were identified through systematic searches of MEDLINE via PubMed, Embase, and the Cochrane Central Register of Controlled Trials (CENTRAL). Our search strategy was based on Medical Subject Headings (MeSH) or Emtree terms and followed the PICOS format: Population (P): patients with AxSpA, including nr-axSpA and AS. Intervention (I): biologics, including TNF-α receptor Fc fusion protein (TNFRFc), TNF-α monoclonal antibodies (TNFmAb), IL17A inhibitor (IL17Ai), IL17A/F dual inhibitor (IL17A/Fi), IL17RAi, JAK inhibitors, including JAK1/3i and JAK1i, IL-6 inhibitor (IL6i), IL-12 and/or IL-23 inhibitor (IL12/23i), and PDE4i, across all treatment durations. Comparison (C): the aforementioned biologics, PLA, and/or sulfasalazine (SSZ). Outcomes (O): clinical response rate and safety. Study design (S): randomized placebo- or active-controlled clinical trials.

We conducted searches from the inception of each database until 20 October 2022 and considered studies published in English. The complete search strategy is provided in Supplementary Table S1. Additionally, we scanned the citations in the included articles to identify studies meeting our inclusion criteria.

### 2.3 Eligibility criteria

We included randomized clinical trials published in peer-reviewed scientific journals. Eligible patients in each study had a documented diagnosis of axial spondyloarthritis (axSpA), which includes two subtypes: AS and nr-axSpA. AS patients met both the Assessment of SpondyloArthritis International Society (ASAS) classification criteria for axSpA (Rudwaleit et al., 2011) and the imaging criterion (sacroiliitis) of the modified New York classification criteria for AS (van der Linden et al., 1984). Nr-axSpA patients met the ASAS classification criteria but did not meet the imaging criterion in the modified New York criteria. Studies recruiting patients with other subforms of axSpA, such as psoriatic arthritis (PsA), reactive arthritis (ReA), and inflammatory bowel disease-associated spondyloarthritis (IBD-SpA), were excluded.

### 2.4 Study selection and data extraction

The retrieved studies were imported into EndNote software (version 20.0). After duplicates were removed, two investigators (Y Yin and E Zhou) independently screened the titles and abstracts to determine the potential of eligibility for inclusion based on the predefined inclusion and exclusion criteria. The full text of the identified studies will be examined. Areas of disagreement or uncertainty were settled by consensus among the investigators. The detailed variables from the eligible studies were extracted. The efficacy outcome measures were ASAS response criteria, including ASAS20 and ASAS40, the improvement of 50% Bath Ankylosing Spondylitis Disease Activity Index (BASDAI50), and Ankylosing Spondylitis Disease Activity Score Inactive Disease (ASDAS-ID). For safety outcomes, treatment-emergent adverse events (TEAEs) were defined as any unfavorable medical occurrence during treatment, regardless of causality. Serious adverse events (SAEs) were defined as TEAEs that resulted in death, hospital admission or prolongation of existing hospital stay, persistent or significant disability, or life-threatening events.

### 2.5 Quality evaluation

We assessed the risk of bias for each included study using the revised Cochrane Risk-of-Bias 2 (Rob2.0) tool (Sterne et al., 2019). The evaluation covered several aspects, including the randomization process, deviations from the intended intervention, missing outcome data, measurement of the outcome, and selection of the reported result. The certainty of the evidence was categorized into three levels: low risk of bias, some concerns, and high risk of bias. Two reviewers independently conducted the bias assessment, and any disagreements were resolved through consensus.

### 2.6 Statistical analysis

We conducted a network meta-analysis using Stata/SE (version 17.0) and R (version 4.2.2), employing a random-effects model. The analysis was based on frequency theory and a multivariate framework. To visualize the comparisons between different interventions, we created evidence network diagrams for various outcome indicators. Consistency testing was performed using both global (Wald test) and local (node-splitting method) approaches within the network (Hoaglin et al., 2011; van Valkenhoef et al., 2016). The global test assessed inconsistency between comparisons, while the local test assessed inconsistency between direct and indirect evidence within each comparison. We calculated summary odds ratios (ORs) with corresponding 95% confidence intervals (95% CIs) for all outcome indicators and presented these estimates in league charts. To assess the potential effectiveness of future trials, we calculated 95% predictive intervals (95% PrIs) of ORs and displayed them on forest plots alongside meta-analysis estimates. To identify interventions with the highest probability of effectiveness, we used the surface under cumulative ranking (SUCRA) curve. SUCRA values, expressed as percentages ranging from 0% to 100%, indicate the probability of achieving the endpoint. We also used a two-dimensional graph to visually assess both efficacy and safety for each intervention. Finally, we employed funnel plots to detect the presence of a small sample effect and assess publication bias in the analysis. Statistical significance was set at *p* < 0.05.

## 3 Results

### 3.1 Search strategy and quality assessment

**FIGURE 1:**
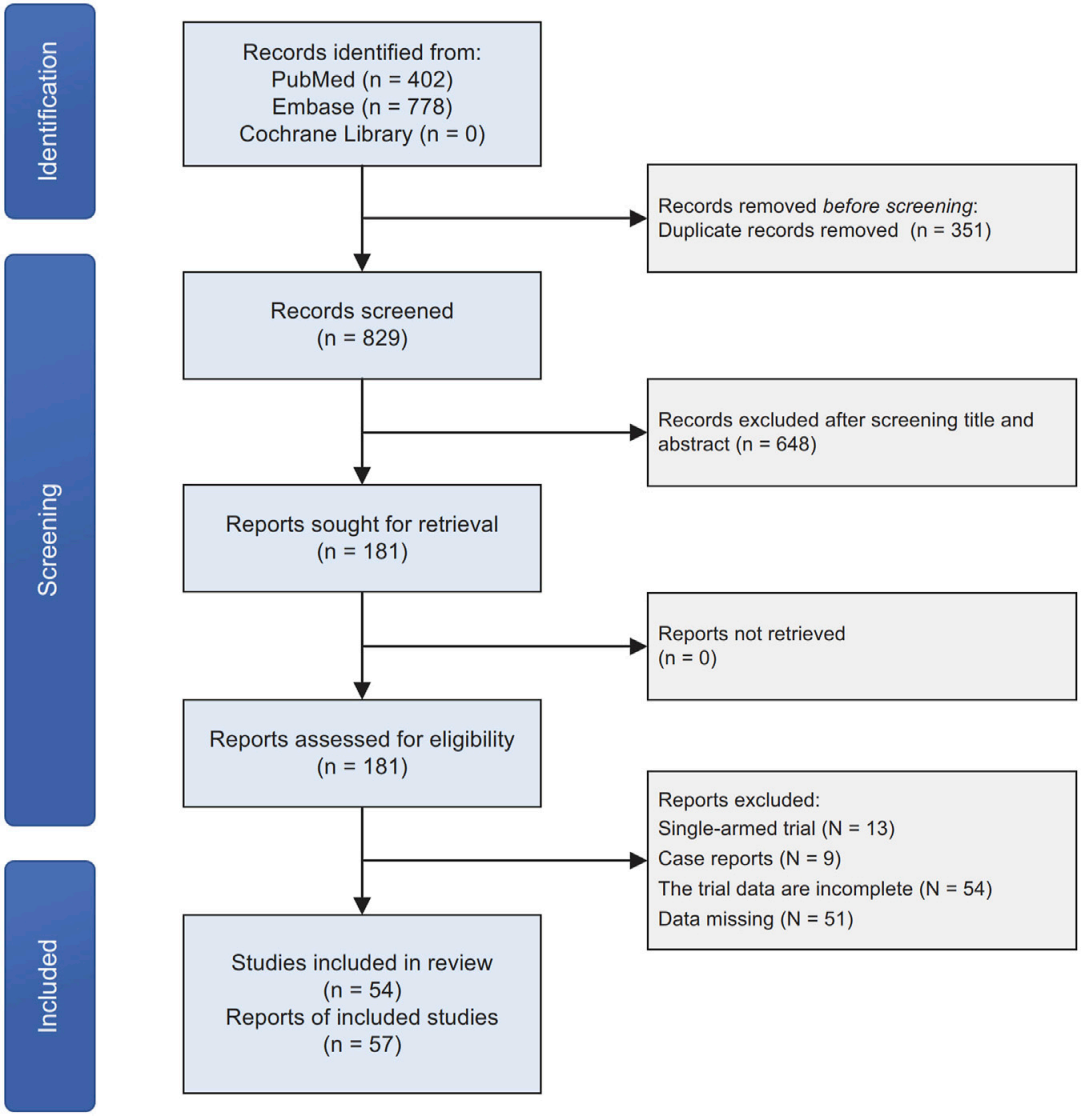
Study selection flowchart depicting the screening process and final included studies.

We initially identified 1,180 original records through our search strategies in electronic databases. After removing 351 duplicates and screening titles and abstracts, 448 articles were excluded. Following a detailed examination of the full text of the remaining 181 publications, 127 studies were excluded. These exclusions were primarily due to the study type being single-armed trials, case reports, or incomplete data. Ultimately, we included 54 articles, encompassing 57 clinical trials, in our quantitative network meta-analysis (Figure 1). The majority of the included studies exhibited a low-to-moderate risk of bias (Supplementary Table S2).

### 3.2 Basic characteristics

The basic characteristics of the included studies are summarized in Table 1. The data represent 57 clinical trials published between 2013 and 2022. A total of 11,787 patients ( 9,057 with AS and 2,730 with nr-axSpA) were recruited and followed for 6–52 weeks. Similar large variations were observed among intervention and control groups for male individuals (ranging from 18.3% to 94.9%) and age (ranging from 31.2 ± 6.6 years to 48.0 ± 10.0 years).

**TABLE 1:** Basic characteristics of the included studies.

Trial and first author	Year	Country	Publication journal	SpA	Intervention	Number	Male	Age^a^	Time point (w)
1. TNFRFc (n = 10)									
ASCEND; Braun et al. (2011)	2011	Germany	*Arthritis Rheum*	AS	Etanercept	379	279	40.7 ± 11.7	16
					Sulfasalazine	187	140	40.9 ± 12.2	
ASCEND; Damjanov et al. (2016)	2016	Serbia	*Rheumatol Int*	AS	Etanercept	190	97	39.4 ± 11.7	16
					Sulfasalazine	149	77	39.1 ± 12.2	
Calin et al. (2004)	*2004*	United Kingdom	*Ann Rheum Dis*	AS	Etanercept	45	36	45.3 ± 9.5	12
					Placebo	39	30	40.7 ± 11.4	
Davis et al. (2003)	2003	United States	*Arthritis Rheum*	AS	Etanercept	138	105	42.1 (24-70)	24
					Placebo	139	105	41.9 (18–65)	
EMBARK; Dougados et al. (2014a)	2014	France	*Arthritis Rheumatol*	Nr-axSpA	Etanercept	106	68	31.9 ± 7.8	12
					Placebo	109	62	32.0 ± 7.8	
EMBARK; Wei et al. (2018)	2016	China	*Int J Rheum Dis*	Nr-axSpA	Etanercept	54	38	32.0 ± 6.8	12
					Placebo	57	36	32.2 ± 8.7	
Song et al. (2011)	2011	Germany	*Ann Rheum Dis*	AxSpA	Etanercept	40	23	34.5 ± 8.6	48
					Sulfasalazine	36	21	32.8 ± 8.4	
SPARSE; Dougados et al. (2014b)	2014	France	*Arthritis Res Ther*	AxSpA	Etanercept	42	24	38.8 ± 12.3	8
					Placebo	48	32	38.9 ± 11.4	
SPINE; Dougados et al. (2011)	2011	France	*Ann Rheum Dis*	AS	Etanercept	39	37	46.0 ± 11.0	12
					Placebo	43	39	48.0 ± 10.0	
van der Heijde et al. (2006a)	2006	The Netherlands	*Ann Rheum Dis*	AS	Etanercept	305	222	41.5 ± 11.0	12
					Placebo	51	40	40.1 ± 10.9	
2. TNFmAb (n = 20)									
ABILITY-1; Sieper et al. (2013)	2013	Germany	*Ann Rheum Dis*	Nr-axSpA	Adalimumab	91	44	37.6 ± 11.3	12
					Placebo	94	40	38.4 ± 10.4	
ABILITY-3; Landewé et al. (2018)	2018	The Netherlands	*Lancet*	Nr-axSpA	Adalimumab	152	96	34.7 ± 10.3	28
					Placebo	153	93	35.3 ± 10.2	
ATLAS; van der Heijde et al. (2006b)	2006	The Netherlands	*Arthritis Rheum*	AS	Adalimumab	208	157	41.7 ± 11.69	24
					Placebo	107	79	43.4 ± 11.32	
Haibel et al. (2008)	2008	Germany	*Arthritis and Rheumatism*	Nr-axSpA	Adalimumab	22	13	38 (25-64)	12
					Placebo	24	12	37 (26–54)	
Horneff et al. (2012)	2012	Germany	*Arthritis Res Ther*	AS	Adalimumab	17	7	15.1 ± 1.5	12
					Placebo	15	8	15.5 ± 1.7	
Huang et al. (2014)	2014	China	*Ann Rheum Dis*	AS	Adalimumab	229	185	30.1 ± 8.7	24
					Placebo	115	95	29.6 ± 7.5	
C-axSpAnd; Deodhar et al. (2019a)	2019	United States	*Arthritis Rheumatol*	Nr-axSpA	Certolizumab	159	78	37.3 ± 10.5	52
					Placebo	158	76	37.4 ± 10.8	
C-OPTIMISE; Landewé et al. (2020)	2020	The Netherlands	*Ann Rheum Dis*	AxSpA	Certolizumab	209	162	32.5 ± 7.1	48
					Placebo	104	19	31.2 ± 6.6	
RAPID-axSpA; Landewé et al. (2014)	2014	The Netherlands	*Ann Rheum Dis*	AxSpA	Certolizumab	218	135	39.1 ± 11.9	12
					Placebo	107	65	39.9 ± 12.4	
Bao et al. (2014)	2014	China	*Rheumatology (Oxford)*	AS	Golimumab	108	90	30.5 ± 10.27	24
					Placebo	105	87	30.6 ± 8.60	
GO-AHEAD; Sieper et al. (2015)	2015	Germany	*Arthritis Rheumatol*	Nr-axSpA	Golimumab	98	61	30.7 ± 67.1	16
					Placebo	100	52	31.7 ± 67.2	
GO-ALIVE; Deodhar et al. (2018)	2018	United States	*J Rheumatol*	AS	Golimumab	105	86	38.4 ± 10.1	16
					Placebo	103	77	39.2 ± 10.8	
GO-RAISE; Inman et al. (2008)	2008	Canada	*Arthritis Rheumatol*	AS	Golimumab	556	400	38.0 (29.0-47.0)	24
					Placebo	78	55	41.0 (31.0–50.0)	
Tam et al. (2014)	2014	China	*Rheumatology (Oxford)*	AS	Golimumab	20	18	35.6 ± 9.93	24
					Placebo	21	19	34.2 ± 10.0	
ASSERT; van der Heijde et al. (2005)	2005	The Netherlands	*Arthritis Rheumatol*	AS	Infliximab	201	157	40.0 (32.0, 47.0)	24
					Placebo	78	68	41.0 (34.0, 47.0)	
Burgos-Vargas et al. (2022)	*2022*	Mexico	*Arthritis Res Ther*	AS	Infliximab	12	12	15.0 ± 1.7	12
					Placebo	*14*	*13*	*14.5 ± 2.7*	
Giardina et al. (2010)	2009	Italy	*Rheumatol Int*	AS	Infliximab	25	19	31.9 ± 9.2	12
					Etanercept	25	20	32.6 ± 6.8	
INFAST; Sieper et al. (2014a)	2014	Germany	*Ann Rheum Dis*	AxSpA	Infliximab	105	72	31.7 ± 8.51	28
					Placebo	51	40	30.7 ± 7.34	
Inman and Maksymowych (2010)	2010	Canada	*J Rheumatol*	AS	Infliximab	39	32	42.9 ± 10.4	12
					Placebo	37	29	39.3 ± 9.0	
Marzo-Ortega et al. (2005)	2005	United Kingdom	*Ann Rheum Dis*	AS	Infliximab	28	23	41 (28-74)	30
					Placebo	14	11	39 (30–56)	
3. IL17Ai (n = 11)									
COAST-V; van der Heijde et al. (2018b)	2018	The Netherlands	*Lancet*	AS	Ixekizumab	164	132	41.2 ± 11.6	16
					Adalimumab	90	73	41.8 ± 11.4	
					Placebo	87	71	42.7 ± 12.0	
COAST-W; Deodhar et al. (2019c)	2019	United States	*Arthritis Rheumatol*	AS	Ixekizumab	212	166	45.8 ± 11.9	16
					Placebo	104	87	46.6 ± 12.7	
COAST-X; Deodhar et al. (2020c)	2020	United States	*Lancet*	Nr-axSpA	Ixekizumab	198	99	40.5 ± 13.4	16
					Placebo	105	44	39.9 ± 12.4	
Erdes et al. (2020)	2020	Russia	*Clin Exp Rheumatol*	AS	Netakimab	66	58	38.0 (35.0-44.0)	16
					Placebo	22	15	15 ± 68.18	
Baeten et al. (2013)	2013	The Netherlands	*Lancet*	AS	Secukinumab	24	14	41.1 ± 10.10	6
					Placebo	6	5	45.0 ± 9.96	
MEASURE 1; Baeten et al. (2015)	2015	The Netherlands	*NEJM*	AS	Secukinumab	249	172	40.2 ± 12.1	16
					Placebo	122	85	43.1 ± 12.4	
MEASURE 2; Baeten et al. (2015)	2015	The Netherlands	*NEJM*	AS	Secukinumab	145	97	42.5 ± 12.8	16
					Placebo	74	56	43.6 ± 13.2	
MEASURE 3; Pavelka et al. (2017)	2017	Czechia	*Arthritis Res Ther*	AS	Secukinumab	150	96	42.5 ± 11.5	16
					Placebo	76	40	42.7 ± 11.4	
MEASURE 4; Kivitz et al. (2018)	2018	United States	*Rheumatol Ther*	AS	Secukinumab	233	164	42.9 ± 11.3	16
					Placebo	117	76	43.4 ± 12.46	
MEASURE 5; Huang et al. (2020)	2020	China	*Chin Med J (Engl)*	AS	Secukinumab	305	252	35.1 ± 10.38	16
					Placebo	153	132	33.0 ± 10.02	
PREVENT; Deodhar et al. (2021a)	2021	United States	*Arthritis Rheumatol*	Nr-axSpA	Secukinumab	369	164	39.5 ± 11.6	16
					Placebo	186	91	39.30 ± 11.47	
4. IL17A/Fi (n = 1)									
BE AGILE; van der Heijde et al. (2020)	2020	The Netherlands	*Ann Rheum Dis*	AS	Bimekizumab	243	207	42.2 ± 11.9	12
					Placebo	60	49	39.7 ± 10.3	
5. IL17RAi (n = 1)									
Wei et al. (2021b)	2021	China	*Ann Rheum Dis*	AxSpA	Brodalumab	80	66	36.6 ± 11.4	16
					Placebo	79	61	38.3 ± 10.8	
6. JAK1/3i (n = 2)									
Deodhar et al. (2021b)	2021	United States	*Ann Rheum Dis*	AS	Tofacitinib	133	116	42.2 ± 11.9	16
					Placebo	136	108	40.0 ± 11.1	
van der Heijde et al. (2017)	2017	The Netherlands	*Ann Rheum Dis*	AS	Tofacitinib	156	111	41.7 ± 11.8	12
					Placebo	51	32	41.9 ± 12.9	
7. JAK1i (n = 4)									
TORTUGA; van der Heijde et al. (2018a)	2018	The Netherlands	*Lancet*	AS	Filgotinib	58	45	41 ± 11.6	12
					Placebo	58	41	42 ± 9.0	
SELECT-AXIS 1; van der Heijde et al. (2019)	2019	The Netherlands	*Lancet*	AS	Upadacitinib	93	63	47.0 ± 12.8	14
					Placebo	94	69	43.7 ± 12.1	
SELECT-AXIS 2 (AS); van der Heijde et al. (2022)	2022	The Netherlands	*Ann Rheum Dis*	AS	Upadacitinib	211	153	42.6 ± 12.4	14
					Placebo	209	158	42.2 ± 11.8	
SELECT-AXIS 2 (nr-axSpA); Deodhar et al. (2022)	2022	United States	*Lancet*	Nr-axSpA	Upadacitinib	156	67	41.6 ± 12.0	14
					Placebo	157	63	42.5 ± 12.4	
8. IL6i (n = 1)									
BUILDER-1; Sieper et al. (2014b)	2014	Germany	*Ann Rheum Dis*	AS	Tocilizumab	51	36	41.6 ± 11.2	12
					Placebo	51	40	42.7 ± 12.6	
9. IL12/23i (n = 4)									
Baeten et al. (2018)	2018	The Netherlands	*Ann Rheum Dis*	AS	Risankizumab	119	88	39.5 ± 10.8	12
					Placebo	40	25	37.6 ± 11.0	
Deodhar (study 1); Deodhar et al. (2019b)	2019	United States	*Arthritis Rheumatol*	AS	Ustekinumab	230	193	39.3 ± 10.9	24
					Placebo	116	101	38.3 ± 11.4	
Deodhar (study 2); Deodhar et al. (2019b)	2019	United States	*Arthritis Rheumatol*	AS	Ustekinumab	211	180	41.5 ± 11.2	24
					Placebo	104	80	40.8 ± 11.7	
Deodhar (study 3); Deodhar et al. (2019b)	2019	United States	*Arthritis Rheumatol*	Nr-axSpA	Ustekinumab	240	116	34.4 ± 8.7	24
					Placebo	116	64	34.0 ± 8.8	
10. PDE4i (n = 2)									
Pathan et al. (2013)	2013	United Kingdom	*Ann Rheum Dis*	AS	Apremilast	17	N/A	44.88 ± 11.1	12
					Placebo	19	N/A	39.21 ± 13.3	
Taylor et al. (2021)	2021	United Kingdom	*J Rheumatol*	AS	Apremilast	326	228	45.0 ± 11.9	24
					Placebo	164	124	44.0 ± 12.9	
11. csDMARD (n = 1)									
Khanna Sharma et al. (2018)	2018	India	*Int J Rheum Dis*	AS	Sulfasalazine	64	N/A	31.32 ± 10.12	24
					Placebo	33	N/A	30.70 ± 8.46	

**AxSpA**, axial spondyloarthritis; **AS**, ankylosing spondylitis; **nr-axSpA**, non-radiographic axial spondyloarthritis; **TNFRFc**, TNFR-Fc fusion protein; **TNFmAb**, TNF-α monoclonal antibody; **IL17Ai**, IL-17A monoclonal antibody; **IL17A/Fi**, IL-17A and IL-17F dual inhibitor; **IL17RA**, IL-17 receptor A monoclonal antibody; **JAK1/3i**, JAK1 and JAK3 inhibitor; **JAK1i**, JAK1 inhibitor; **IL6i**, IL-6 inhibitor; **IL12/23i**, IL-12 and/or IL-23 inhibitor; **PDE4i**, phosphodiesterase-4 inhibitor; **csDMARD**, conventional synthetic disease-modifying antirheumatic drug.

^a^ Mean with SD of age was preferred where available; otherwise, range or median age was used.

**FIGURE 2:**
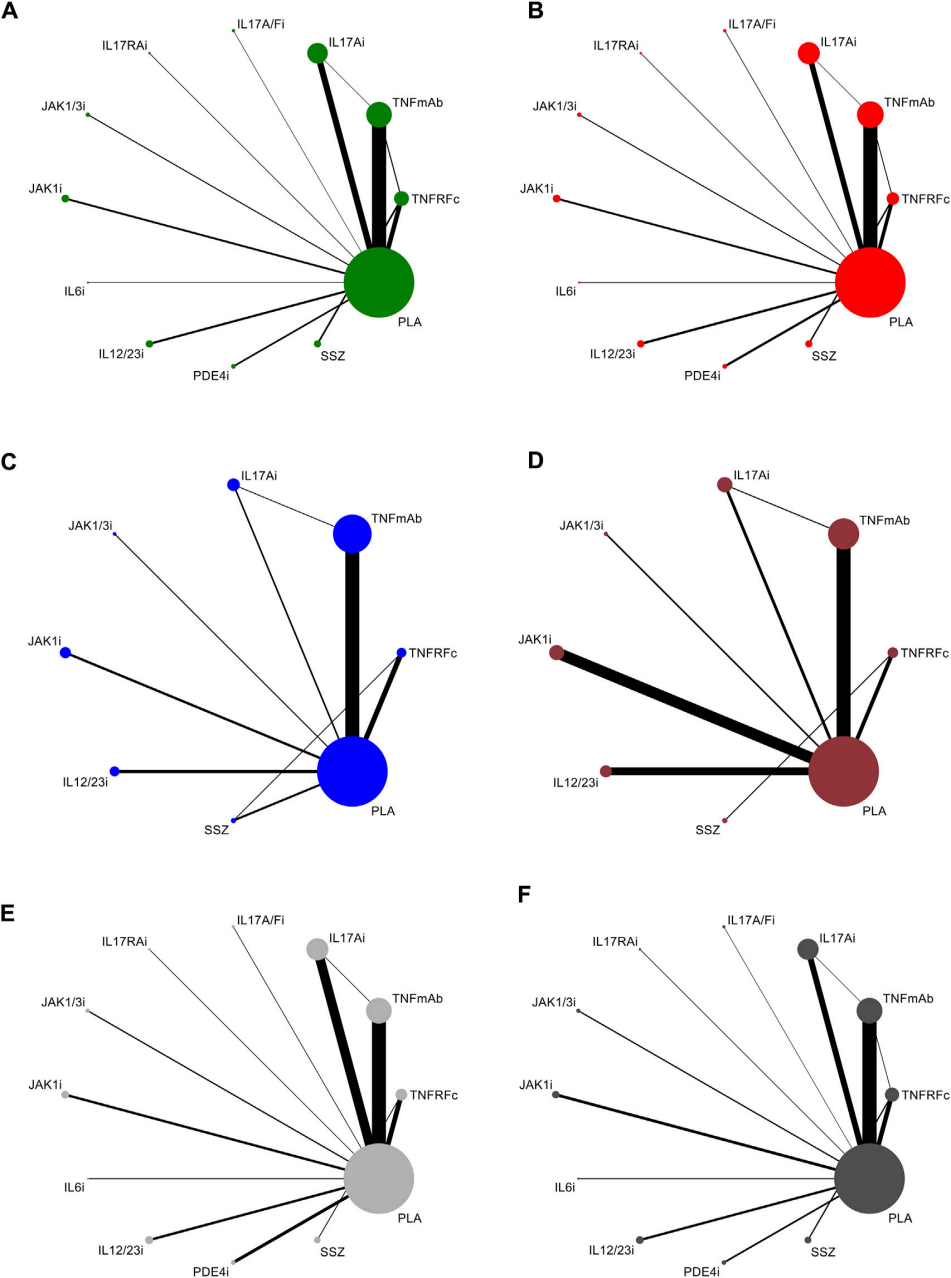
Evidence network plots for the analysis of **(A)** ASAS20, **(B)** ASAS40, **(C)** BASDAI50, **(D)** ASDAS-ID, **(E)** TEAEs, and **(F)** SAEs. Line thickness corresponds to the number of trials comparing each pair of treatments. Node size is proportional to the number of randomized participants receiving the treatment. TNFRFc, TNFR-Fc fusion protein; TNFmAb, TNF-α monoclonal antibody; IL17Ai, IL-17A monoclonal antibody; IL17A/Fi, IL-17A and IL-17F dual inhibitor; IL17RA, IL-17 receptor A monoclonal antibody; JAK1/3i, JAK1 and JAK3 inhibitor; JAK1i, JAK1 inhibitor; IL6i, IL-6 inhibitor; IL12/23i, IL-12 and/or IL-23 inhibitor; PDE4i, phosphodiesterase-4 inhibitor; PLA: placebo.

All articles involved biologics, including TNFRFc [10 studies involving etanercept (Davis et al., 2003; Calin et al., 2004; van der Heijde et al., 2006a; Braun et al., 2011; Dougados et al., 2011; Song et al., 2011; Dougados et al., 2014a; Dougados et al., 2014b; Damjanov et al., 2016; Wei et al., 2018)], TNFmAb [six studies involving adalimumab (van der Heijde et al., 2006b; Haibel et al., 2008; Horneff et al., 2012; Sieper et al., 2013; Huang et al., 2014; Landewé et al., 2018), three studies involving certolizumab (Landewé et al., 2014; Deodhar et al., 2019a; Landewé et al., 2020), five studies involving golimumab (Inman et al., 2008; Bao et al., 2014; Tam et al., 2014; Sieper et al., 2015; Deodhar et al., 2018), and six studies involving infliximab (Marzo-Ortega et al., 2005; van der Heijde et al., 2005; Giardina et al., 2010; Inman and Maksymowych, 2010; Sieper et al., 2014a; Burgos-Vargas et al., 2022)], IL17Ai [three studies involving ixekizumab (van der Heijde et al., 2018b; Deodhar et al., 2019c; Deodhar et al., 2020c), one study involving netakimab (Erdes et al., 2020), and seven studies involving secukinumab (Baeten et al., 2013; Baeten et al., 2015; Pavelka et al., 2017; Kivitz et al., 2018; Huang et al., 2020; Deodhar et al., 2021a)], IL17A/Fi [one study involving bimekizumab (van der Heijde et al., 2020)], IL17RAi [one study involving brodalumab (Wei et al., 2021b)], IL6i [one study involving tocilizumab (Sieper et al., 2014b)], IL12/23i [one study involving risankizumab (Baeten et al., 2018) and three studies involving ustekinumab (Deodhar et al., 2019b)], and PDE4i [two studies involving apremilast (Pathan et al., 2013; Taylor et al., 2021)], small-molecule drugs, including JAK1/3i [two studies involving tofacitinib (van der Heijde et al., 2017; Deodhar et al., 2021b)] and JAK1i [one study involving filgotinib (van der Heijde et al., 2018a) and three studies involving upadacitinib (van der Heijde et al., 2019; Deodhar et al., 2022; van der Heijde et al., 2022)], and csDMARD [one study involving SSZ (Khanna Sharma et al., 2018)]. All studies included at least one outcome measure for comparison. The network plots of outcomes to exhibit all the available evidence of each treatment are displayed in Figure 2.

### 3.3 Efficacy analysis

**FIGURE 3:**
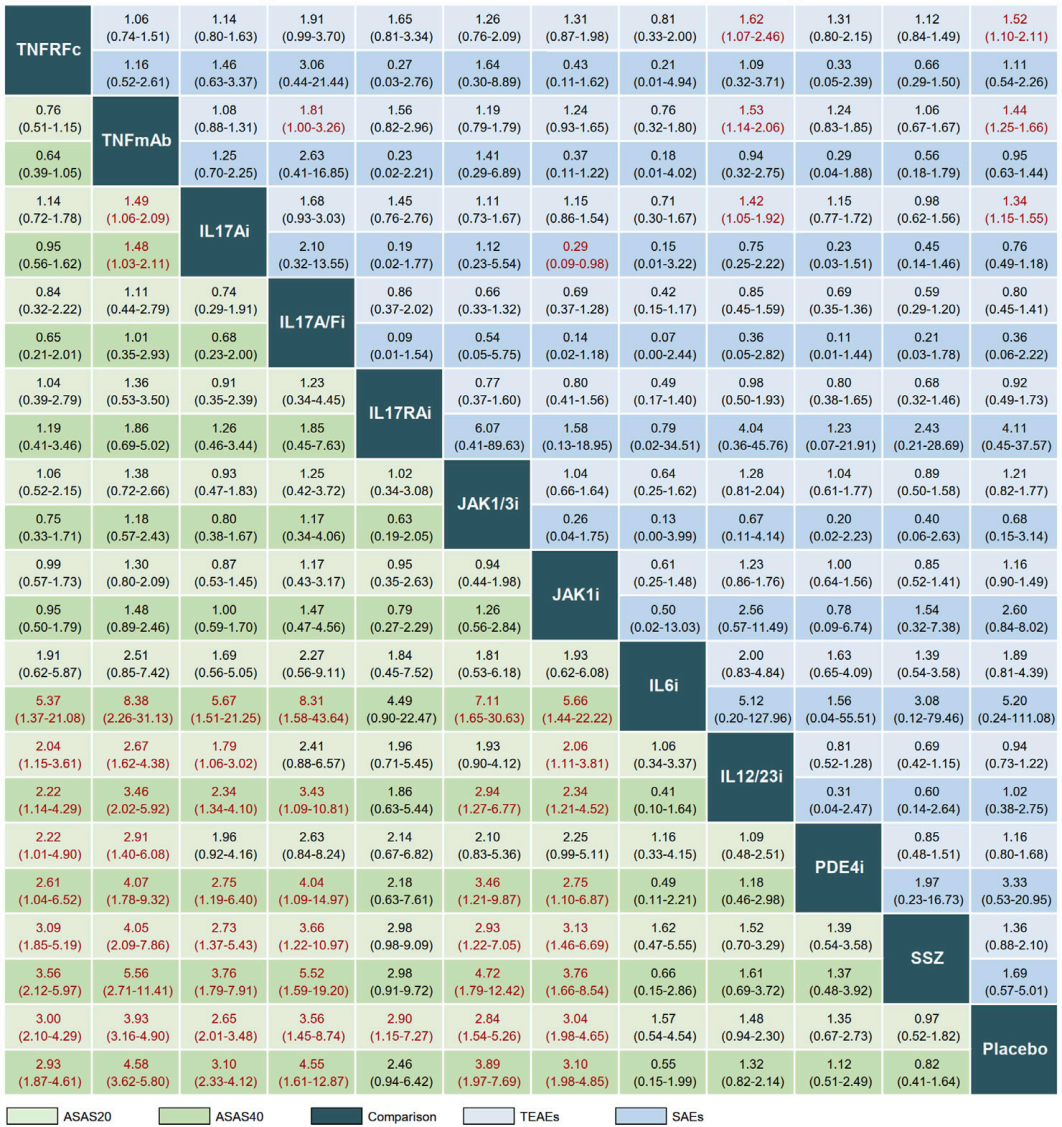
League plot comparing efficacy and safety across interventions. Treatment comparisons should be read from left to right. Efficacy data are presented as odds ratios with 95% confidence intervals. Values above 1 favor the column-defining treatment. TNFRFc, TNFR-Fc fusion protein; TNFmAb, TNF-α monoclonal antibody; IL17Ai, IL-17A monoclonal antibody; IL17A/Fi, IL-17A and IL-17F dual inhibitor; IL17RA, IL-17 receptor A monoclonal antibody; JAK1/3i, JAK1 and JAK3 inhibitor; JAK1i, JAK1 inhibitor; IL6i, IL-6 inhibitor; IL12/23i, IL-12 and/or IL-23 inhibitor; PDE4i, phosphodiesterase-4 inhibitor; PLA, placebo.

The league plot in Figure 3 illustrates the relative efficacy of different treatments. When compared to PLA, seven treatments showed significantly greater efficacy in achieving an ASAS20 response: TNFRFc (OR, 3.00; 95% CI, 2.10–4.29), TNFmAb (OR, 3.93; 95% CI, 3.16–4.90), IL17Ai (OR, 2.65; 95% CI, 2.01–3.48), IL17A/Fi (OR, 3.56; 95% CI, 1.45–8.74), IL17RAi (OR, 2.90; 95% CI, 1.15–7.27), JAK1/3i (OR, 2.84; 95% CI, 1.54–5.26), and JAK1i (OR, 3.04; 95% CI, 1.98–4.65). Regarding head-to-head comparisons, statistically significant improvements in achieving ASAS20 response were observed in comparisons such as TNFRFc or TNFmAb vs. IL12/23i, PDE4i, or SSZ; IL17Ai or JAK1i vs. IL12/23i or SSZ; and IL17A/Fi or JAK1/3i vs. SSZ (Figure 3).

In terms of ASAS40, significant differences in clinical response were observed after treatment with six drugs (TNFRFc, TNFmAb, IL17Ai, IL17A/Fi, JAK1/3i, and JAK1i) in comparison with PLA. The better clinical efficacy in achieving ASAS40 response were achieved by TNFRFc, TNFmAb, IL17Ai, IL17A/Fi, JAK1/3i, and JAK1i compared to IL6i, IL12/23i, PDE4i, SSZ, or PLA (Figure 3).

As for BASDAI50, there are four treatments (TNFRFc, TNFmAb, IL17Ai, and JAK1i) that showed better response rates compared to PLA, and head-to-head comparison indicates that three (TNFRFc, TNFmAb, and IL17Ai) of these four treatments are effective compared to IL12/23i; similar results are obtained in the evaluation of ASDAS-ID response (Supplementary Figure S1). The forest plots of the relative mean effects of treatments, along with 95% CIs and 95% PrIs, are shown in Supplementary Figure S2.

**FIGURE 4:**
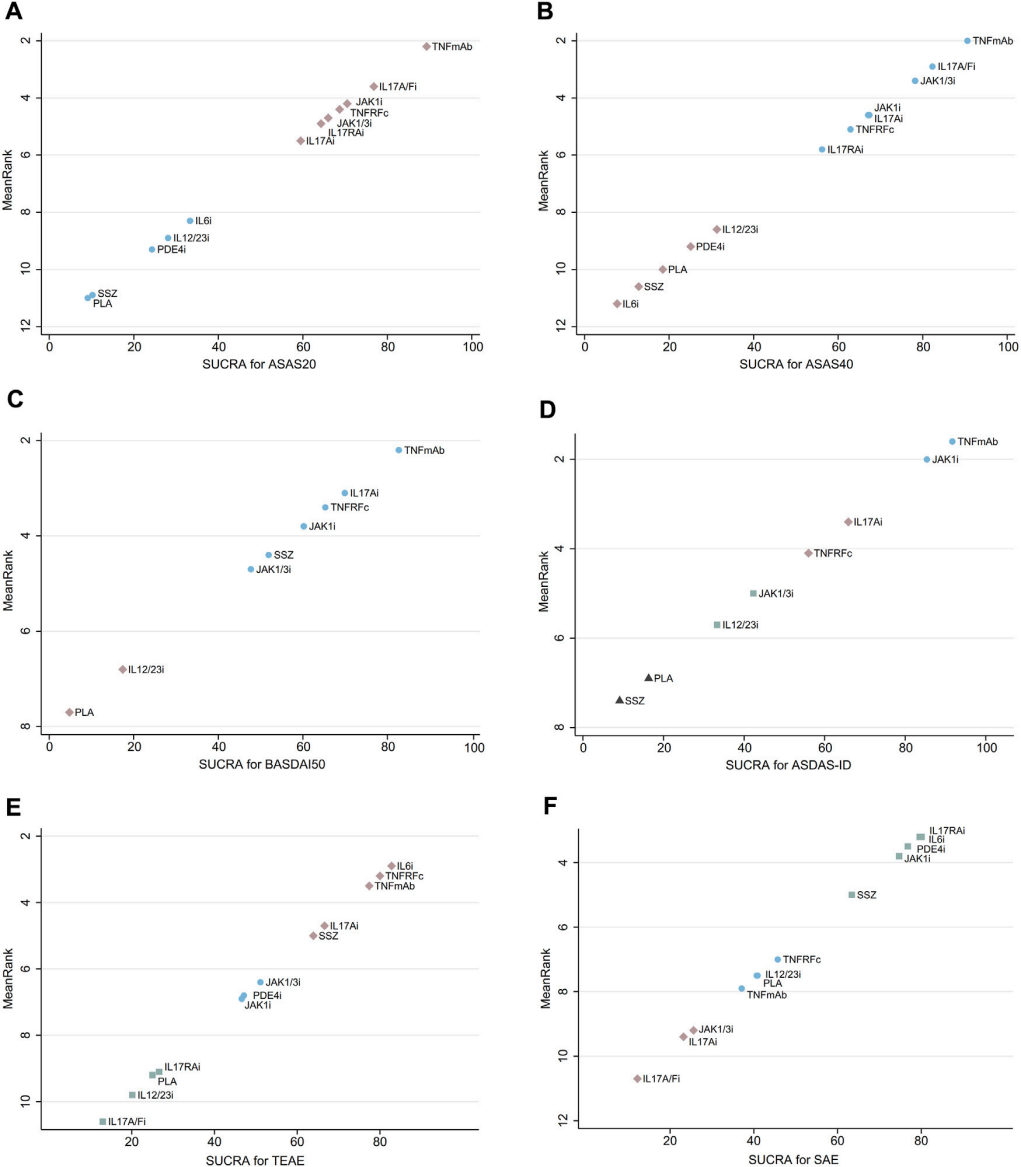
SUCRA ranking plots for **(A)** ASAS20, **(B)** ASAS40, **(C)** BASDAI50, **(D)** ASDAS-ID, **(E)** TEAEs, and **(F)** SAEs. Treatments located toward the upper right corner exhibit the most favorable ranking for that outcome compared to other options. TNFRFc, TNFR-Fc fusion protein; TNFmAb, TNF-α monoclonal antibody; IL17Ai, IL-17A monoclonal antibody; IL17A/Fi, IL-17A and IL-17F dual inhibitor; IL17RA, IL-17 receptor A monoclonal antibody; JAK1/3i, JAK1 and JAK3 inhibitor; JAK1i, JAK1 inhibitor; IL6i, IL-6 inhibitor; IL12/23i, IL-12 and/or IL-23 inhibitor; PDE4i, phosphodiesterase-4 inhibitor; PLA, placebo.

According to the SUCRA-based relative ranking of treatments, TNFmAb (SUCRA, 89.3%) had the highest probability to achieve ASAS20 response, and the efficacy of the remaining treatments were ranked from high to low in the following order: IL17A/Fi (SUCRA, 76.8%) > JAK1i (SUCRA, 70.5%) > TNFRFc (SUCRA, 68.7%) > JAK1/3i (SUCRA, 66.0%) > IL17RAi (SUCRA, 64.3%) > IL17Ai (SUCRA, 59.5%) > IL6i (SUCRA, 33.3%) > IL12/23i (SUCRA, 28.1%) > PDE4i (SUCRA, 24.3%) > SSZ (SUCRA, 10.2%) > PLA (SUCRA, 9.1%) (Figure 4). In the following analysis, TNFmAb still ranked the highest probability for achieving efficacy in ASAS40, BASDAI50, and ASDAS-ID (Figure 4). The detailed ranking plots for a single outcome using probabilities are shown in Supplementary Figure S3.

### 3.4 Subgroup analysis

Because two categories of patients were included, we evaluated whether the efficacy outcomes of drugs varied in different patient populations (AS and nr-axSpA). Considering efficacy of both ASAS20 and ASAS40 responses, six treatments (TNFRFc, TNFmAb, IL17Ai, IL17A/Fi, JAK1/3i, and JAK1i) and four treatments (TNFRFc, TNFmAb, IL17Ai, and JAK1i) were more effective than PLA in patients with AS and nr-axSpA, respectively; other treatments (IL6i, IL12/23i, PDE4i, and SSZ) had no effect in these patients, being similar to the results in axSpA patients (Supplementary Figures S5, S6). TNFmAb was ranked the most effective treatment for patients with AS; this result was also found in patients with nr-axSpA (Supplementary Figure S7). Note that IL12/23i (OR, 1.54; 95% CI, 1.03–2.29) had a higher ASAS20 response than PLA in patients with AS. In the original article, three studies recruiting patients with nr-axSpA were prematurely discontinued due to failure in receiving endpoints in a concurrent study (Deodhar et al., 2019b). Therefore, these data should be interpreted with caution.

### 3.5 Safety analysis

A total of 49 and 55 articles reported the occurrence of TEAEs and SAEs, respectively. Our results showed that TNFRFc (OR, 1.52; 95% CI, 1.10–2.11), TNFmAb (OR, 1.44; 95% CI, 1.25–1.66), and IL17Ai (OR, 1.34; 95% CI, 1.15–1.55) had a higher incidence of increasing risk of TEAEs compared with PLA. Additionally, TNFmAb had a higher risk of TEAEs compared to IL17A/Fi (OR, 1.81; 95% CI, 1.00–3.26). For the analysis of SAEs, the overwhelming majority of treatments showed no significant advantage or disadvantage compared to PLA or among each other, and only IL17Ai treatment had a lower risk of SAEs compared with JAK1i (OR, 0.29; 95% CI, 0.09–0.98) (Figure 3). The forest plots of the relative mean effects of treatments are shown in Supplementary Figure S2. A lower incidence of TEAEs and SAEs was observed in patients treated with IL17A/Fi (SUCRA, 10.6) and IL17RAi (SUCRA, 10.7), respectively, compared to those undergoing other treatments (Figure 4).

**FIGURE 5:**
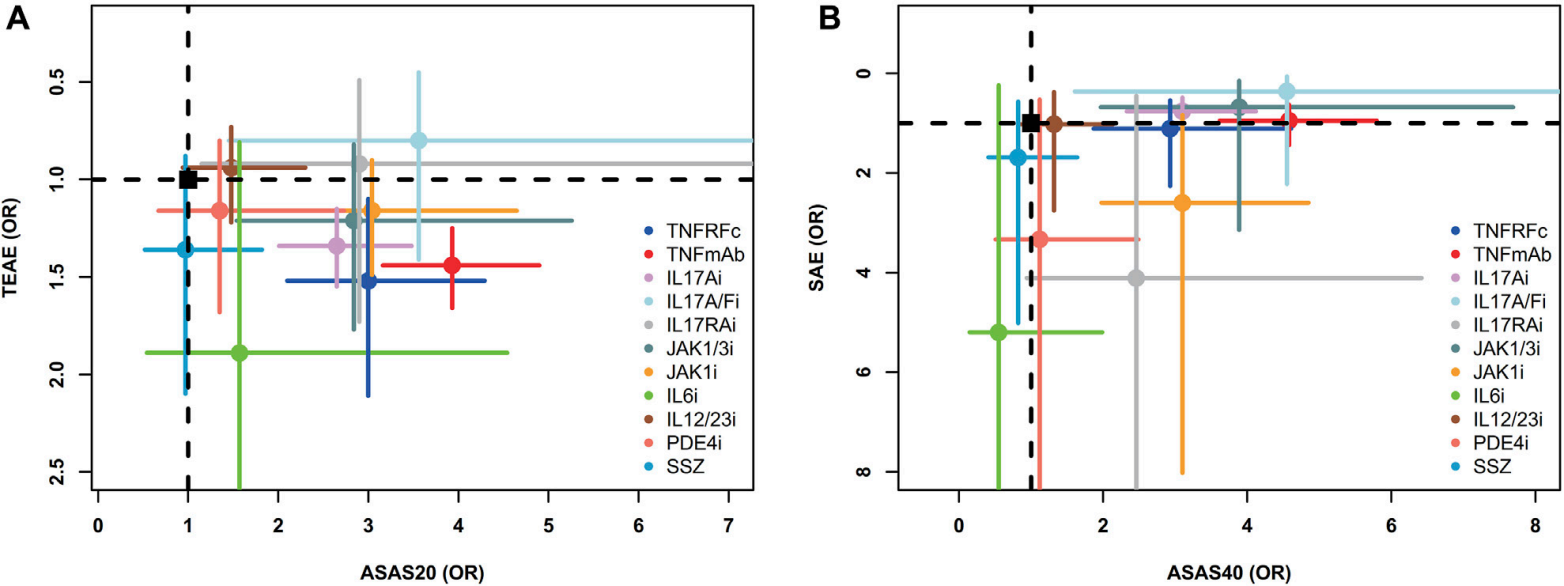
Two-dimensional graphs for **(A)** TEAEs *versus* ASAS20 and **(B)** SAEs *versus* ASAS40. Individual treatments are nodes, with placebo as a black square. Data are mean odds ratios with error bars representing 95% confidence intervals. Nodes in the upper right corner indicate treatments with high efficacy and low adverse events. TNFRFc, TNFR-Fc fusion protein; TNFmAb, TNF-α monoclonal antibody; IL17Ai, IL-17A monoclonal antibody; IL17A/Fi, IL-17A and IL-17F dual inhibitor; IL17RA, IL-17 receptor A monoclonal antibody; JAK1/3i, JAK1 and JAK3 inhibitor; JAK1i, JAK1 inhibitor; IL6i, IL-6 inhibitor; IL12/23i, IL-12 and/or IL-23 inhibitor; PDE4i, phosphodiesterase-4 inhibitor; PLA, placebo.

Two-dimensional graphs were illustrated to evaluate the overall performance (Figure 5). For the comprehensive assessment using ASAS20 and TEAEs, IL17A/Fi might be the best choice in balancing efficacy and safety. Similar results were also observed in the comprehensive assessment using ASAS40 and SAEs (Figure 5).

### 3.6 Inconsistency and publication bias

There was no global inconsistency for most outcomes except for BASDAI50 (χ^2^, 11.78; *p* = 0.0082) in our results (Supplementary Table S3). The local inconsistency test implied that there was no difference between most of the direct comparison and indirect comparison, except for ASAS40 (TNFmAb vs. IL17Ai and IL17Ai vs. PLA) and BASDAI50 (TNFRFc vs. SSZ, TNFRFc vs. PLA, and SSZ vs. PLA), which suggests low overall inconsistency (Supplementary Table S4). Comparison-adjusted funnel plots were used to examine publication bias. No significant visual asymmetry was found in the plots of the efficacy and safety outcomes, showing no obvious publication bias among the aforementioned analyses (Supplementary Figure S8).

## 4 Discussion

The primary objective in treating axSpA is to enhance long-term health-related quality of life (Ramiro et al., 2022). The introduction of biologics, followed by the release of small-molecule drugs, has played a crucial role in achieving this objective (Ramiro et al., 2022). While various types of these drugs have been approved and have shown clear efficacy in these patients, their differing performance in clinical response rates and potential adverse events have garnered significant attention. Therefore, a comprehensive assessment of various treatment regimens may be beneficial for clinicians when selecting the most appropriate treatment for these patients.

Our network meta-analysis provides the most comprehensive summary to date by comparing the efficacy and safety of 11 classes of biologics and small-molecule drugs in patients with axSpA. Furthermore, this study offers the first insights into the relative efficacy of these drugs in nr-axSpA patients. The results indicate that seven treatments (TNFmAb, IL17A/Fi, JAK1i, TNFRFc, JAK1/3i, IL17RAi, and IL17Ai) were associated with superior clinical response compared to PLA. Among them, TNFmAb demonstrated the best response across all efficacy outcomes included in this study. Safety analyses suggested that IL17A/Fi might carry the lowest risk of TEAEs and SAEs. TNFmAb had the third highest SUCRA value for TEAEs, suggesting that its remarkable efficacy might be accompanied by a slightly higher rate of adverse events. Finally, most treatments showed no significant advantage or disadvantage regarding SAEs.

Several scholars have attempted comparative comparisons of treatment efficacy in ankylosing spondylitis (Deodhar et al., 2020a; Cao et al., 2022). Deodhar et al. (2020a) evaluated the relative efficacy of four types of biologics (IL17Ai, JAK inhibitors, TNF inhibitors, and PDE4i) across 28 interventions in 30 included studies. Their study identified tofacitinib (JAK1/3i) as the top-ranked treatment for ASAS20 response, followed by golimumab (TNFmAb) and filgotinib (JAK1i). However, safety outcomes were not evaluated in this study. Results from the study by Cao et al. (2022) showed the highest ASAS20 and ASAS40 response rates in patients treated with IL17A/Fi. In our study, IL17A/Fi was ranked the second highest for these clinical response rates among active treatments, which differs slightly from this finding. These discrepancies may be attributed to the broader scope of our study, which included both AS and nr-axSpA patients, incorporated more recently published trials (e.g., PDE4i and JAK1i), and evaluated more promisingly effective drugs (e.g., IL17RAi) for treating axSpA, compared to previous analyses. Regarding safety, no significant increase in the risk of SAEs was observed for any of the drugs compared to PLA, consistent with previous studies (Betts et al., 2016; Deodhar et al., 2020a; Cao et al., 2022; Lee, 2022).

Nr-axSpA is considered to represent an early stage of AS or just an abortive form of axSpA (Baraliakos and Braun, 2015). Correspondingly, patients with nr-axSpA are less likely to be treated with biologics (Hunter et al., 2021). Registry and clinical trial data suggest that patients with AS and nr-axSpA exhibit similar clinical manifestations, disease activity, disease burden, and treatment needs, regardless of the presence of radiographic damage (Rudwaleit et al., 2009; López-Medina et al., 2019). Currently, few biologics have been approved for managing nr-axSpA (Deodhar et al., 2020b; Ramiro et al., 2022). Several other drugs are used for these patients, but off-label. Another novel finding of this study is that TNFmAb also ranked the highest for efficacy outcomes in patients with nr-axSpA. These findings could serve as a reference for the development of further management recommendations and the approval of additional drugs in this field.

## 5 Limitations

This study has several limitations. First, drugs with the same mechanism of action were grouped together for analysis regardless of molecular structure differences, which may not fully reflect the heterogeneity in efficacy. Second, concomitant medications like NSAIDs and csDMARDs were allowed in some included trials, which could influence results. However, baseline medication use was balanced between arms within each trial. Together with the consistent results from inconsistency and publication bias assessments, the relative treatment effects observed in this analysis are considered reliable. Third, patients across a wide range of blinded periods from 6 to 52 weeks were analyzed together, precluding conclusions about specific time points. However, these findings still provide meaningful evidence regarding axSpA treatment, especially in the short-to-medium term. Longer follow-up is necessary to fully evaluate rare adverse events like malignancy. Therefore, while informative for clinical decision-making, the results should be interpreted judiciously considering the study limitations.

## 6 Conclusion

This network meta-analysis evaluated the efficacy and safety of various biologics and small-molecule drugs in patients with axSpA. Our findings suggest that TNFmAb may provide the greatest efficacy based on the outcomes assessed, while IL17A/Fi was associated with the relatively lowest risk and had the best performance in balancing efficacy and safety. Clinicians should discuss the balance between benefit and harm with individual patients when considering treatment options.

## Data Availability

The original contributions presented in the study are included in the article/Supplementary Material; further inquiries can be directed to the corresponding author.

## References

[B1] Baeten D. Baraliakos X. Braun J. Sieper J. Emery P. Van Der Heijde D. (2013). Anti-interleukin-17A monoclonal antibody secukinumab in treatment of ankylosing spondylitis: a randomised, double-blind, placebo-controlled trial. Lancet 382, 1705–1713. 10.1016/S0140-6736(13)61134-4 24035250

[B2] Baeten D. Østergaard M. Wei J. C. Sieper J. Järvinen P. Tam L. S. (2018). Risankizumab, an IL-23 inhibitor, for ankylosing spondylitis: results of a randomised, double-blind, placebo-controlled, proof-of-concept, dose-finding phase 2 study. Ann. Rheum. Dis. 77, 1295–1302. 10.1136/annrheumdis-2018-213328 29945918 PMC6104676

[B3] Baeten D. Sieper J. Braun J. Baraliakos X. Dougados M. Emery P. (2015). Secukinumab, an interleukin-17a inhibitor, in ankylosing spondylitis. N. Engl. J. Med. 373, 2534–2548. 10.1056/NEJMoa1505066 26699169

[B4] Bao C. Huang F. Khan M. A. Fei K. Wu Z. Han C. (2014). Safety and efficacy of golimumab in Chinese patients with active ankylosing spondylitis: 1-year results of a multicentre, randomized, double-blind, placebo-controlled phase III trial. Rheumatol. Oxf. 53, 1654–1663. 10.1093/rheumatology/keu132 24729398

[B5] Baraliakos X. Braun J. (2015). Non-radiographic axial spondyloarthritis and ankylosing spondylitis: what are the similarities and differences? RMD Open 1, e000053. 10.1136/rmdopen-2015-000053 26557375 PMC4632143

[B6] Betts K. A. Griffith J. Song Y. Mittal M. Joshi A. Wu E. Q. (2016). Network meta-analysis and cost per responder of tumor necrosis factor-α and interleukin inhibitors in the treatment of active ankylosing spondylitis. Rheumatol. Ther. 3, 323–336. 10.1007/s40744-016-0038-y 27747581 PMC5127962

[B7] Braun J. Van Der Horst-Bruinsma I. E. Huang F. Burgos-Vargas R. Vlahos B. Koenig A. S. (2011). Clinical efficacy and safety of etanercept versus sulfasalazine in patients with ankylosing spondylitis: a randomized, double-blind trial. Arthritis Rheum. 63, 1543–1551. 10.1002/art.30223 21630245

[B8] Burgos-Vargas R. Loyola-Sanchez A. Ramiro S. Reding-Bernal A. Alvarez-Hernandez E. Van Der Heijde D. (2022). A randomized, double-blind, placebo-controlled 12-week trial of infliximab in patients with juvenile-onset spondyloarthritis. Arthritis Res. Ther. 24, 187. 10.1186/s13075-022-02877-9 35941676 PMC9358905

[B9] Calin A. Dijkmans B. A. Emery P. Hakala M. Kalden J. Leirisalo-Repo M. (2004). Outcomes of a multicentre randomised clinical trial of etanercept to treat ankylosing spondylitis. Ann. Rheum. Dis. 63, 1594–1600. 10.1136/ard.2004.020875 15345498 PMC1754832

[B10] Cantini F. Niccoli L. Nannini C. Cassarà E. Kaloudi O. Giulio Favalli E. (2017). Second-line biologic therapy optimization in rheumatoid arthritis, psoriatic arthritis, and ankylosing spondylitis. Semin. Arthritis Rheum. 47, 183–192. 10.1016/j.semarthrit.2017.03.008 28413099

[B11] Cao Z. Guo J. Li Q. Li Y. Wu J. (2022). Optimal biologic drugs for the treatment of ankylosing spondylitis: results from a network meta-analysis and network metaregression. Biomed. Res. Int. 2022, 8316106. 10.1155/2022/8316106 35845959 PMC9279076

[B12] Caso F. Costa L. Triggianese P. Maione F. Bertolini N. Vastarella M. (2023). Recent developments for new investigational JAK inhibitors in psoriatic arthritis. Expert Opin. Investig. Drugs 32, 361–371. 10.1080/13543784.2023.2207737 37096862

[B13] Damjanov N. Shehhi W. A. Huang F. Kotak S. Burgos-Vargas R. Shirazy K. (2016). Assessment of clinical efficacy and safety in a randomized double-blind study of etanercept and sulfasalazine in patients with ankylosing spondylitis from Eastern/Central Europe, Latin America, and Asia. Rheumatol. Int. 36, 643–651. 10.1007/s00296-016-3452-0 26968844

[B14] Danve A. Deodhar A. (2022). Treatment of axial spondyloarthritis: an update. Nat. Rev. Rheumatol. 18, 205–216. 10.1038/s41584-022-00761-z 35273385

[B15] Davis J. C. Jr. Van Der Heijde D. Braun J. Dougados M. Cush J. Clegg D. O. (2003). Recombinant human tumor necrosis factor receptor (etanercept) for treating ankylosing spondylitis: a randomized, controlled trial. Arthritis Rheum. 48, 3230–3236. 10.1002/art.11325 14613288

[B16] Deodhar A. Blanco R. Dokoupilová E. Hall S. Kameda H. Kivitz A. J. (2021a). Improvement of signs and symptoms of nonradiographic axial spondyloarthritis in patients treated with secukinumab: primary results of a randomized, placebo-controlled phase III study. Arthritis Rheumatol. 73, 110–120. 10.1002/art.41477 32770640 PMC7839589

[B17] Deodhar A. Chakravarty S. D. Cameron C. Peterson S. Hensman R. Fogarty S. (2020a). A systematic review and network meta-analysis of current and investigational treatments for active ankylosing spondylitis. Clin. Rheumatol. 39, 2307–2315. 10.1007/s10067-020-04970-3 32107666 PMC7338808

[B18] Deodhar A. Gensler L. S. Kay J. Maksymowych W. P. Haroon N. Landewé R. (2019a). A fifty-two-week, randomized, placebo-controlled trial of certolizumab pegol in nonradiographic axial spondyloarthritis. Arthritis Rheumatol. 71, 1101–1111. 10.1002/art.40866 30848558 PMC6619287

[B19] Deodhar A. Gensler L. S. Sieper J. Clark M. Calderon C. Wang Y. (2019b). Three multicenter, randomized, double-blind, placebo-controlled studies evaluating the efficacy and safety of ustekinumab in axial spondyloarthritis. Arthritis Rheumatol. 71, 258–270. 10.1002/art.40728 30225992

[B20] Deodhar A. Poddubnyy D. Pacheco-Tena C. Salvarani C. Lespessailles E. Rahman P. (2019c). Efficacy and safety of ixekizumab in the treatment of radiographic axial spondyloarthritis: sixteen-week results from a phase III randomized, double-blind, placebo-controlled trial in patients with prior inadequate response to or intolerance of tumor necrosis factor inhibitors. Arthritis Rheumatol. 71, 599–611. 10.1002/art.40753 30343531 PMC6593790

[B21] Deodhar A. Reveille J. D. Harrison D. D. Kim L. Lo K. H. Leu J. H. (2018). Safety and efficacy of golimumab administered intravenously in adults with ankylosing spondylitis: results through week 28 of the GO-ALIVE study. J. Rheumatol. 45, 341–348. 10.3899/jrheum.170487 29247154

[B22] Deodhar A. Sandoval D. Holdsworth E. Booth N. Hunter T. (2020b). Use and switching of biologic therapy in patients with non-radiographic axial spondyloarthritis: a patient and provider survey in the United States. Rheumatol. Ther. 7, 415–423. 10.1007/s40744-020-00208-5 32328928 PMC7211225

[B23] Deodhar A. Sliwinska-Stanczyk P. Xu H. Baraliakos X. Gensler L. S. Fleishaker D. (2021b). Tofacitinib for the treatment of ankylosing spondylitis: a phase III, randomised, double-blind, placebo-controlled study. Ann. Rheum. Dis. 80, 1004–1013. 10.1136/annrheumdis-2020-219601 33906853 PMC8292568

[B24] Deodhar A. Van Den Bosch F. Poddubnyy D. Maksymowych W. P. Van Der Heijde D. Kim T. H. (2022). Upadacitinib for the treatment of active non-radiographic axial spondyloarthritis (SELECT-AXIS 2): a randomised, double-blind, placebo-controlled, phase 3 trial. Lancet 400, 369–379. 10.1016/S0140-6736(22)01212-0 35908570

[B25] Deodhar A. Van Der Heijde D. Gensler L. S. Kim T. H. Maksymowych W. P. Østergaard M. (2020c). Ixekizumab for patients with non-radiographic axial spondyloarthritis (COAST-X): a randomised, placebo-controlled trial. Lancet 395, 53–64. 10.1016/S0140-6736(19)32971-X 31813637

[B26] Dougados M. Braun J. Szanto S. Combe B. Elbaz M. Geher P. (2011). Efficacy of etanercept on rheumatic signs and pulmonary function tests in advanced ankylosing spondylitis: results of a randomised double-blind placebo-controlled study (SPINE). Ann. Rheum. Dis. 70, 799–804. 10.1136/ard.2010.139261 21317434 PMC3070274

[B27] Dougados M. Van Der Heijde D. Sieper J. Braun J. Maksymowych W. P. Citera G. (2014a). Symptomatic efficacy of etanercept and its effects on objective signs of inflammation in early nonradiographic axial spondyloarthritis: a multicenter, randomized, double-blind, placebo-controlled trial. Arthritis Rheumatol. 66, 2091–2102. 10.1002/art.38721 24891317

[B28] Dougados M. Wood E. Combe B. Schaeverbeke T. Miceli-Richard C. Berenbaum F. (2014b). Evaluation of the nonsteroidal anti-inflammatory drug-sparing effect of etanercept in axial spondyloarthritis: results of the multicenter, randomized, double-blind, placebo-controlled SPARSE study. Arthritis Res. Ther. 16, 481. 10.1186/s13075-014-0481-5 25428762 PMC4282738

[B29] Erdes S. Nasonov E. Kunder E. Pristrom A. Soroka N. Shesternya P. (2020). Primary efficacy of netakimab, a novel interleukin-17 inhibitor, in the treatment of active ankylosing spondylitis in adults. Clin. Exp. Rheumatol. 38, 27–34. 31025924

[B30] Giardina A. R. Ferrante A. Ciccia F. Impastato R. Miceli M. C. Principato A. (2010). A 2-year comparative open label randomized study of efficacy and safety of etanercept and infliximab in patients with ankylosing spondylitis. Rheumatol. Int. 30, 1437–1440. 10.1007/s00296-009-1157-3 19851772

[B31] Haibel H. Rudwaleit M. Listing J. Heldmann F. Wong R. L. Kupper H. (2008). Efficacy of adalimumab in the treatment of axial spondylarthritis without radiographically defined sacroiliitis: results of a twelve-week randomized, double-blind, placebo-controlled trial followed by an open-label extension up to week fifty-two. Arthritis Rheum. 58, 1981–1991. 10.1002/art.23606 18576337

[B32] Hoaglin D. C. Hawkins N. Jansen J. P. Scott D. A. Itzler R. Cappelleri J. C. (2011). Conducting indirect-treatment-comparison and network-meta-analysis studies: report of the ISPOR task force on indirect treatment comparisons good research practices: part 2. Value Health 14, 429–437. 10.1016/j.jval.2011.01.011 21669367

[B33] Horneff G. Fitter S. Foeldvari I. Minden K. Kuemmerle-Deschner J. Tzaribacev N. (2012). Double-blind, placebo-controlled randomized trial with adalimumab for treatment of juvenile onset ankylosing spondylitis (JoAS): significant short term improvement. Arthritis Res. Ther. 14, R230. 10.1186/ar4072 23095307 PMC3580542

[B34] Huang F. Gu J. Zhu P. Bao C. Xu J. Xu H. (2014). Efficacy and safety of adalimumab in Chinese adults with active ankylosing spondylitis: results of a randomised, controlled trial. Ann. Rheum. Dis. 73, 587–594. 10.1136/annrheumdis-2012-202533 23475983

[B35] Huang F. Sun F. Wan W. G. Wu L. J. Dong L. L. Zhang X. (2020). Secukinumab provided significant and sustained improvement in the signs and symptoms of ankylosing spondylitis: results from the 52-week, Phase III China-centric study, MEASURE 5. Chin. Med. J. Engl. 133, 2521–2531. 10.1097/CM9.0000000000001099 32925287 PMC7722578

[B36] Hunter T. Sandoval D. Booth N. Holdsworth E. Deodhar A. (2021). Comparing symptoms, treatment patterns, and quality of life of ankylosing spondylitis and non-radiographic axial spondyloarthritis patients in the USA: findings from a patient and rheumatologist Survey. Clin. Rheumatol. 40, 3161–3167. 10.1007/s10067-021-05642-6 33611647 PMC8289774

[B37] Hutton B. Salanti G. Caldwell D. M. Chaimani A. Schmid C. H. Cameron C. (2015). The PRISMA extension statement for reporting of systematic reviews incorporating network meta-analyses of health care interventions: checklist and explanations. Ann. Intern Med. 162, 777–784. 10.7326/M14-2385 26030634

[B38] Inman R. D. Davis J. C. Jr. Heijde D. Diekman L. Sieper J. Kim S. I. (2008). Efficacy and safety of golimumab in patients with ankylosing spondylitis: results of a randomized, double-blind, placebo-controlled, phase III trial. Arthritis Rheum. 58, 3402–3412. 10.1002/art.23969 18975305

[B39] Inman R. D. Maksymowych W. P. CANDLE Study Group (2010). A double-blind, placebo-controlled trial of low dose infliximab in ankylosing spondylitis. J. Rheumatol. 37, 1203–1210. 10.3899/jrheum.091042 20231198

[B40] Khanna Sharma S. Kadiyala V. Naidu G. Dhir V. (2018). A randomized controlled trial to study the efficacy of sulfasalazine for axial disease in ankylosing spondylitis. Int. J. Rheum. Dis. 21, 308–314. 10.1111/1756-185X.13124 28737251

[B41] Kivitz A. J. Wagner U. Dokoupilova E. Supronik J. Martin R. Talloczy Z. (2018). Efficacy and safety of secukinumab 150 mg with and without loading regimen in ankylosing spondylitis: 104-week results from MEASURE 4 study. Rheumatol. Ther. 5, 447–462. 10.1007/s40744-018-0123-5 30121827 PMC6251842

[B42] Landewé R. Braun J. Deodhar A. Dougados M. Maksymowych W. P. Mease P. J. (2014). Efficacy of certolizumab pegol on signs and symptoms of axial spondyloarthritis including ankylosing spondylitis: 24-week results of a double-blind randomised placebo-controlled Phase 3 study. Ann. Rheum. Dis. 73, 39–47. 10.1136/annrheumdis-2013-204231 24013647 PMC3888598

[B43] Landewé R. B. Van Der Heijde D. Dougados M. Baraliakos X. Van Den Bosch F. E. Gaffney K. (2020). Maintenance of clinical remission in early axial spondyloarthritis following certolizumab pegol dose reduction. Ann. Rheum. Dis. 79, 920–928. 10.1136/annrheumdis-2019-216839 32381562 PMC7307216

[B44] Landewé R. Sieper J. Mease P. Inman R. D. Lambert R. G. Deodhar A. (2018). Efficacy and safety of continuing versus withdrawing adalimumab therapy in maintaining remission in patients with non-radiographic axial spondyloarthritis (ABILITY-3): a multicentre, randomised, double-blind study. Lancet 392, 134–144. 10.1016/S0140-6736(18)31362-X 29961640

[B45] Lawson D. O. Eraso M. Mbuagbaw L. Joanes M. Aves T. Leenus A. (2021). Tumor necrosis factor inhibitor dose reduction for axial spondyloarthritis: a systematic review and meta-analysis of randomized controlled trials. Arthritis Care Res. Hob. 73, 861–872. 10.1002/acr.24184 32166872

[B46] Lee Y. H. (2022). Comparative efficacy and safety of janus kinase inhibitors and secukinumab in patients with active ankylosing spondylitis: a systematic review and meta-analysis. Pharmacology 107, 537–544. 10.1159/000525627 35817017 PMC9811419

[B47] Li S. Li F. Mao N. Wang J. Xie X. (2022). Efficacy and safety of Janus kinase inhibitors in patients with ankylosing spondylitis: a systematic review and meta-analysis. Eur. J. Intern Med. 102, 47–53. 10.1016/j.ejim.2022.04.007 35461744

[B48] Li T. Puhan M. A. Vedula S. S. Singh S. Dickersin K. Ad Hoc Network Meta-analysis Methods Meeting Workin g Group (2011). Network meta-analysis-highly attractive but more methodological research is needed. BMC Med. 9, 79. 10.1186/1741-7015-9-79 21707969 PMC3159133

[B49] López-Medina C. Ramiro S. Van Der Heijde D. Sieper J. Dougados M. Molto A. (2019). Characteristics and burden of disease in patients with radiographic and non-radiographic axial Spondyloarthritis: a comparison by systematic literature review and meta-analysis. RMD Open 5, e001108. 10.1136/rmdopen-2019-001108 31803500 PMC6890393

[B50] Marzo-Ortega H. Mcgonagle D. Jarrett S. Haugeberg G. Hensor E. O'connor P. (2005). Infliximab in combination with methotrexate in active ankylosing spondylitis: a clinical and imaging study. Ann. Rheum. Dis. 64, 1568–1575. 10.1136/ard.2004.022582 15829577 PMC1755262

[B51] Pathan E. Abraham S. Van Rossen E. Withrington R. Keat A. Charles P. J. (2013). Efficacy and safety of apremilast, an oral phosphodiesterase 4 inhibitor, in ankylosing spondylitis. Ann. Rheum. Dis. 72, 1475–1480. 10.1136/annrheumdis-2012-201915 22984171

[B52] Pavelka K. Kivitz A. Dokoupilova E. Blanco R. Maradiaga M. Tahir H. (2017). Efficacy, safety, and tolerability of secukinumab in patients with active ankylosing spondylitis: a randomized, double-blind phase 3 study, MEASURE 3. Arthritis Res. Ther. 19, 285. 10.1186/s13075-017-1490-y 29273067 PMC5741872

[B53] Ramiro S. Nikiphorou E. Sepriano A. Ortolan A. Webers C. Baraliakos X. (2022). Response to: correspondence on "ASAS-EULAR recommendations for the management of axial spondyloarthritis: 2022 update" by Braun *et al* . Ann. Rheum. Dis. 82, e206. 10.1136/ard-2023-223937 36878690

[B54] Rudwaleit M. Haibel H. Baraliakos X. Listing J. Märker-Hermann E. Zeidler H. (2009). The early disease stage in axial spondylarthritis: results from the German Spondyloarthritis Inception Cohort. Arthritis Rheum. 60, 717–727. 10.1002/art.24483 19248087

[B55] Rudwaleit M. Van Der Heijde D. Landewé R. Akkoc N. Brandt J. Chou C. T. (2011). The Assessment of SpondyloArthritis international Society classification criteria for peripheral spondyloarthritis and for spondyloarthritis in general. Ann. Rheumatic Dis. 70, 25–31. 10.1136/ard.2010.133645 21109520

[B56] Sieper J. Lenaerts J. Wollenhaupt J. Rudwaleit M. Mazurov V. I. Myasoutova L. (2014a). Efficacy and safety of infliximab plus naproxen versus naproxen alone in patients with early, active axial spondyloarthritis: results from the double-blind, placebo-controlled INFAST study, Part 1. Ann. Rheum. Dis. 73, 101–107. 10.1136/annrheumdis-2012-203201 23696633 PMC3888606

[B57] Sieper J. Poddubnyy D. (2017). Axial spondyloarthritis. Lancet 390, 73–84. 10.1016/S0140-6736(16)31591-4 28110981

[B58] Sieper J. Porter-Brown B. Thompson L. Harari O. Dougados M. (2014b). Assessment of short-term symptomatic efficacy of tocilizumab in ankylosing spondylitis: results of randomised, placebo-controlled trials. Ann. Rheum. Dis. 73, 95–100. 10.1136/annrheumdis-2013-203559 23765873 PMC3888605

[B59] Sieper J. Van Der Heijde D. Dougados M. Maksymowych W. P. Scott B. B. Boice J. A. (2015). A randomized, double-blind, placebo-controlled, sixteen-week study of subcutaneous golimumab in patients with active nonradiographic axial spondyloarthritis. Arthritis Rheumatol. 67, 2702–2712. 10.1002/art.39257 26139307 PMC4755041

[B60] Sieper J. Van Der Heijde D. Dougados M. Mease P. J. Maksymowych W. P. Brown M. A. (2013). Efficacy and safety of adalimumab in patients with non-radiographic axial spondyloarthritis: results of a randomised placebo-controlled trial (ABILITY-1). Ann. Rheum. Dis. 72, 815–822. 10.1136/annrheumdis-2012-201766 22772328 PMC3664374

[B61] Song I. H. Hermann K. Haibel H. Althoff C. E. Listing J. Burmester G. (2011). Effects of etanercept versus sulfasalazine in early axial spondyloarthritis on active inflammatory lesions as detected by whole-body MRI (ESTHER): a 48-week randomised controlled trial. Ann. Rheum. Dis. 70, 590–596. 10.1136/ard.2010.139667 21372193 PMC3211465

[B62] Sterne J. a.C. Savović J. Page M. J. Elbers R. G. Blencowe N. S. Boutron I. (2019). RoB 2: a revised tool for assessing risk of bias in randomised trials. Bmj 366, l4898. 10.1136/bmj.l4898 31462531

[B63] Sunzini F. D'antonio A. Fatica M. Triggianese P. Conigliaro P. Greco E. (2022). What's new and what's next for biological and targeted synthetic treatments in psoriatic arthritis? Expert Opin. Biol. Ther. 22, 1545–1559. 10.1080/14712598.2022.2152321 36453200

[B64] Tam L. S. Shang Q. Kun E. W. Lee K. L. Yip M. L. Li M. (2014). The effects of golimumab on subclinical atherosclerosis and arterial stiffness in ankylosing spondylitis—a randomized, placebo-controlled pilot trial. Rheumatol. Oxf. 53, 1065–1074. 10.1093/rheumatology/ket469 24501241

[B65] Taylor P. C. Van Der Heijde D. Landewé R. Mccue S. Cheng S. Boonen A. (2021). A phase III randomized study of apremilast, an oral phosphodiesterase 4 inhibitor, for active ankylosing spondylitis. J. Rheumatol. 48, 1259–1267. 10.3899/jrheum.201088 33589554

[B66] Van Der Heijde D. Baraliakos X. Gensler L. S. Maksymowych W. P. Tseluyko V. Nadashkevich O. (2018a). Efficacy and safety of filgotinib, a selective Janus kinase 1 inhibitor, in patients with active ankylosing spondylitis (TORTUGA): results from a randomised, placebo-controlled, phase 2 trial. Lancet 392, 2378–2387. 10.1016/S0140-6736(18)32463-2 30360970

[B67] Van Der Heijde D. Baraliakos X. Sieper J. Deodhar A. Inman R. D. Kameda H. (2022). Efficacy and safety of upadacitinib for active ankylosing spondylitis refractory to biological therapy: a double-blind, randomised, placebo-controlled phase 3 trial. Ann. Rheum. Dis. 81, 1515–1523. 10.1136/ard-2022-222608 35788492 PMC9606523

[B68] Van Der Heijde D. Cheng-Chung Wei J. Dougados M. Mease P. Deodhar A. Maksymowych W. P. (2018b). Ixekizumab, an interleukin-17A antagonist in the treatment of ankylosing spondylitis or radiographic axial spondyloarthritis in patients previously untreated with biological disease-modifying anti-rheumatic drugs (COAST-V): 16 week results of a phase 3 randomised, double-blind, active-controlled and placebo-controlled trial. Lancet 392, 2441–2451. 10.1016/S0140-6736(18)31946-9 30360964

[B69] Van Der Heijde D. Da Silva J. C. Dougados M. Geher P. Van Der Horst-Bruinsma I. Juanola X. (2006a). Etanercept 50 mg once weekly is as effective as 25 mg twice weekly in patients with ankylosing spondylitis. Ann. Rheum. Dis. 65, 1572–1577. 10.1136/ard.2006.056747 16968715 PMC1798458

[B70] Van Der Heijde D. Deodhar A. Wei J. C. Drescher E. Fleishaker D. Hendrikx T. (2017). Tofacitinib in patients with ankylosing spondylitis: a phase II, 16-week, randomised, placebo-controlled, dose-ranging study. Ann. Rheum. Dis. 76, 1340–1347. 10.1136/annrheumdis-2016-210322 28130206 PMC5738601

[B71] Van Der Heijde D. Dijkmans B. Geusens P. Sieper J. Dewoody K. Williamson P. (2005). Efficacy and safety of infliximab in patients with ankylosing spondylitis: results of a randomized, placebo-controlled trial (ASSERT). Arthritis Rheum. 52, 582–591. 10.1002/art.20852 15692973

[B72] Van Der Heijde D. Gensler L. S. Deodhar A. Baraliakos X. Poddubnyy D. Kivitz A. (2020). Dual neutralisation of interleukin-17A and interleukin-17F with bimekizumab in patients with active ankylosing spondylitis: results from a 48-week phase IIb, randomised, double-blind, placebo-controlled, dose-ranging study. Ann. Rheum. Dis. 79, 595–604. 10.1136/annrheumdis-2020-216980 32253184 PMC7213320

[B73] Van Der Heijde D. Kivitz A. Schiff M. H. Sieper J. Dijkmans B. A. Braun J. (2006b). Efficacy and safety of adalimumab in patients with ankylosing spondylitis: results of a multicenter, randomized, double-blind, placebo-controlled trial. Arthritis Rheum. 54, 2136–2146. 10.1002/art.21913 16802350

[B74] Van Der Heijde D. Song I. H. Pangan A. L. Deodhar A. Van Den Bosch F. Maksymowych W. P. (2019). Efficacy and safety of upadacitinib in patients with active ankylosing spondylitis (SELECT-AXIS 1): a multicentre, randomised, double-blind, placebo-controlled, phase 2/3 trial. Lancet 394, 2108–2117. 10.1016/S0140-6736(19)32534-6 31732180

[B75] Van Der Linden S. Valkenburg H. A. Cats A. (1984). Evaluation of diagnostic criteria for ankylosing spondylitis. A proposal for modification of the New York criteria. Arthritis Rheum. 27, 361–368. 10.1002/art.1780270401 6231933

[B76] Van Valkenhoef G. Dias S. Ades A. E. Welton N. J. (2016). Automated generation of node-splitting models for assessment of inconsistency in network meta-analysis. Res. Synth. Methods 7, 80–93. 10.1002/jrsm.1167 26461181 PMC5057346

[B77] Webers C. Ortolan A. Sepriano A. Falzon L. Baraliakos X. Landewé R. B. M. (2022). Efficacy and safety of biological DMARDs: a systematic literature review informing the 2022 update of the ASAS-EULAR recommendations for the management of axial spondyloarthritis. Ann. Rheum. Dis. 82, 130–141. 10.1136/ard-2022-223298 36270657

[B78] Wei J.C.-C. Kim T.-H. Kishimoto M. Ogusu N. Jeong H. Kobayashi S. (2021a). Efficacy and safety of brodalumab, an anti-IL17RA monoclonal antibody, in patients with axial spondyloarthritis: 16-week results from a randomised, placebo-controlled, phase 3 trial. Ann. Rheumatic Dis. 80, 1014–1021. 10.1136/annrheumdis-2020-219406 PMC8292606 33827787

[B79] Wei J. C. Kim T. H. Kishimoto M. Ogusu N. Jeong H. Kobayashi S. (2021b). Efficacy and safety of brodalumab, an anti-IL17RA monoclonal antibody, in patients with axial spondyloarthritis: 16-week results from a randomised, placebo-controlled, phase 3 trial. Ann. Rheum. Dis. 80, 1014–1021. 10.1136/annrheumdis-2020-219406 33827787 PMC8292606

[B80] Wei J. C. Tsai W. C. Citera G. Kotak S. Llamado L. (2018). Efficacy and safety of etanercept in patients from Latin America, Central Europe and Asia with early non-radiographic axial spondyloarthritis. Int. J. Rheum. Dis. 21, 1443–1451. 10.1111/1756-185X.12973 27863065

[B81] Yin Y. Wang M. Liu M. Zhou E. Ren T. Chang X. (2020). Efficacy and safety of IL-17 inhibitors for the treatment of ankylosing spondylitis: a systematic review and meta-analysis. Arthritis Res. Ther. 22, 111. 10.1186/s13075-020-02208-w 32398096 PMC7216398

