# Supplementary material for: Comparison of biologics and small-molecule drugs in axial spondyloarthritis: a systematic review and network meta-analysis
Source: Front Pharmacol. 2023 Oct 24;14:1226528. doi: 10.3389/fphar.2023.1226528 (PMC10628508; doi:10.3389/fphar.2023.1226528)
Supplement: Supplementary file 1 [file DataSheet1.DOCX]

Supplementary Material

Comparison of Biologics and Small Molecule Drugs in Axial Spondyloarthritis: A Systematic Review and Network Meta-analysis

Yufeng Yin¹, Mingjun Wang¹, Michun He¹, Erye Zhou¹*, Jian Wu¹*

*** Correspondence:** Corresponding Author: eryezhou@163.com (Erye Zhou); njwujian@163.com (Jian Wu)

## Supplementary Table 1. Electronic search strategy

| **I. Pubmed** | **#1 Population:**  (("Spondylitis, Ankylosing"[Mesh] OR "Non-Radiographic Axial Spondyloarthritis"[Mesh] OR "Spondylarthritis"[Mesh]) AND "Humans"[Mesh]) NOT "Arthritis, Psoriatic"[Mesh]  **#2 Intervention and Comparison:**  "Tumor Necrosis Factor Inhibitors"[Mesh] OR "Etanercept" [Supplementary Concept] OR "Adalimumab" [Supplementary Concept] OR "Certolizumab Pegol" [Supplementary Concept] OR "Golimumab" [Supplementary Concept] OR "Infliximab" [Supplementary Concept] OR "Ixekizumab" [Supplementary Concept] OR "Netakimab" [Supplementary Concept] OR "Secukinumab" [Supplementary Concept] OR "Bimekizumab" [Supplementary Concept] OR "Brodalumab" [Supplementary Concept] OR "Janus Kinase Inhibitors"[Mesh] OR "Tofacitinib" [Supplementary Concept] OR "GLPG0634" [Supplementary Concept] OR "Upadacitinib" [Supplementary Concept] OR "Tocilizumab" [Supplementary Concept] OR "Risankizumab" [Supplementary Concept] OR "Phosphodiesterase 4 Inhibitors"[Mesh] OR "Apremilast" [Supplementary Concept] OR "Antirheumatic Agents"[Mesh] OR "Sulfasalazine"[Mesh] OR "Antirheumatic Agents"[Mesh]  **#3 Outcome:**  "Treatment Outcome"[Mesh]  **#4 Study design:**  "Randomized Controlled Trial" [Publication Type] OR "Controlled Clinical Trial" [Publication Type] OR "Randomized Controlled Trials as Topic"[Mesh] OR "Controlled Clinical Trials as Topic"[Mesh] OR "Random Allocation"[Mesh] OR "Double-Blind Method"[Mesh] OR "Clinical Trial" [Publication Type] OR "Clinical Trials as Topic"[Mesh]  **#1 AND #2 AND #3 AND #4** |
| --- | --- |
| **II. Embase** | **Population:** (('ankylosing spondylitis'/exp OR 'Bechterew disease' OR 'ankylating spondylitis' OR 'ankylopoietic spondylarthritis' OR 'ankylopoietic spondylitis' OR 'ankylosing spine' OR 'ankylosing spondilitis' OR 'ankylosing spondylarthritis' OR 'ankylosing spondylarthrosis' OR 'ankylosing spondylitis' OR 'ankylosis spondylitis' OR 'ankylotic spondylitis' OR 'bekhterev disease' OR 'morbus bechterew' OR 'spinal ankylosis' OR 'spine ankylosis' OR 'spondylarthritis ankylopoietica' OR 'spondylarthritis ankylosans' OR 'spondylarthrosis ankylopoietica' OR 'spondylitis ankylopoetica' OR 'spondylitis ankylopoietica' OR 'spondylitis, ankylosing' OR 'spondyloarthritis ankylopoietica' OR 'vertebral ankylosis') OR ('non-radiographic axial spondyloarthritis'/exp OR 'Nr-axSpA (spondyloarthritis)' OR 'non-radiographic axial spondyloarthritis') OR spondylarthritis/exp OR (spondylarthritis/exp OR 'arthritis, spine' OR 'spine arthritis' OR 'spondylarthritis' OR 'spondyloarthritis' OR 'vertebral arthritis' OR 'vertebral osteo-arthritis' OR 'vertebral osteoarthritis')) AND **Intervention:** (('tumor necrosis factor inhibitor'/exp OR 'TNF alpha inhibitor' OR 'TNF inhibitor' OR 'anti TNF agent' OR 'anti TNF alpha agent' OR 'anti tumor necrosis factor agent' OR 'anti tumour necrosis factor agent' OR 'tumor necrosis factor alpha inhibitor' OR 'tumor necrosis factor inhibitor' OR 'tumor necrosis factor inhibitors' OR 'tumour necrosis factor alpha inhibitor' OR 'tumour necrosis factor inhibitor') OR ('interleukin 17 antibody'/exp OR 'il 17 antibody' OR 'interleukin 17 antibody') OR ('Janus kinase inhibitor'/exp OR 'JAK inhibitor' OR 'Janus kinase inhibitor' OR 'Janus kinase inhibitors' OR 'Janus tyrosine kinase inhibitor') OR 'interleukin 6 receptor blocking agent'/exp OR 'interleukin 12 inhibitor'/exp OR 'Interleukin 23 inhibitor' OR ('phosphodiesterase IV inhibitor'/exp OR 'PDE 4 inhibitor' OR 'PDE 4 inhibitors' OR 'PDE IV inhibitor' OR 'PDE IV inhibitors' OR 'PDE type 4 inhibitor' OR 'PDE type 4 inhibitors' OR 'PDE type IV inhibitor' OR 'PDE type IV inhibitors' OR 'PDE4 inhibitor' OR 'PDE4 inhibitors' OR 'phosphodiesterase 4 inhibitor' OR 'phosphodiesterase 4 inhibitors' OR 'phosphodiesterase IV inhibitor' OR 'phosphodiesterase IV inhibitors' OR 'phosphodiesterase type 4 inhibitor' OR 'phosphodiesterase type 4 inhibitors' OR 'phosphodiesterase type IV inhibitor' OR 'phosphodiesterase type IV inhibitors')) AND **Comparison:** ((placebo/exp OR 'placebo' OR 'placebo gel' OR 'placebos') OR 'disease modifying antirheumatic drug'/exp) AND **Outcome:** ('efficacy parameters'/exp OR (safety/exp OR 'safety' OR 'safety management' OR 'safety precaution' OR 'safety protection' OR 'safety regulation')) AND **Study design:** ('randomized controlled trial'/br OR 'controlled trial, randomized' OR 'randomised controlled study' OR 'randomised controlled trial' OR 'randomized controlled study' OR 'randomized controlled trial' OR 'trial, randomized controlled') |
| **Cochrane Central Register of Controlled Trials (CENTRAL)** | **'Population** ( "Ankylosing Spondylitis" OR "Non-radiographic Axial Spondylitis" OR "Arthritis Of Spine" ) AND **Intervention** ( "Tumour Necrosis Factor Inhibitors" OR "Interleukin-17 Inhibitor" OR "Interleukin-23 Inhibitor" OR "Interleukin-12/23 Inhibitor" OR "Janus kinase inhibitor" OR "Interleukin-6" OR "Synthetic Disease-Modifying Antirheumatic Drug" OR "Phosphodiesterase 4 inhibitor") AND **Comparison** ( "Placebo" OR "Sulfasalazine" OR "Conventional Synthetic Disease-Modifying Antirheumatic Drug" OR "Tumour Necrosis Factor Inhibitors" OR "Interleukin-17 Inhibitor" OR "Interleukin-23 Inhibitor" OR "Interleukin-12/23 Inhibitor" OR "Janus kinase inhibitor" OR "Phosphodiesterase 4 inhibitor")' AND **Outcome** ( "Clinical effectiveness" OR "Efficacy" OR "Patient safety event" ) |

## Supplementary Table 2. Methodological quality and risk of bias


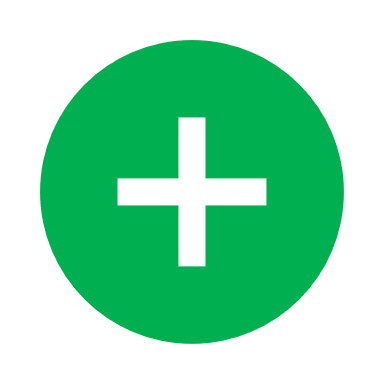
Low risk of bias
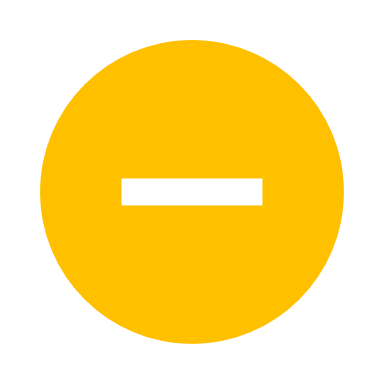
Some concerns
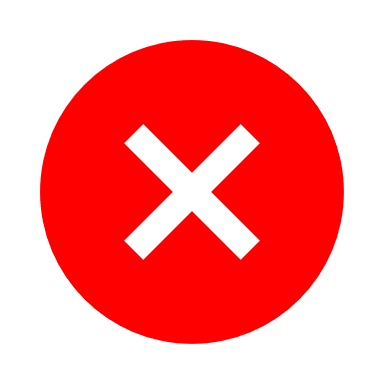
High risk of bias

| **The trial and first author** | **Randomisation process** | **Deviations from the intended intervention** | **Missing outcome data** | **Measurement of the outcome** | **Selection of the reported result** |
| --- | --- | --- | --- | --- | --- |
| **1. TNFRFcFP (n = 10)** |  |  |  |  |  |
| ASCEND (Braun)(1) | 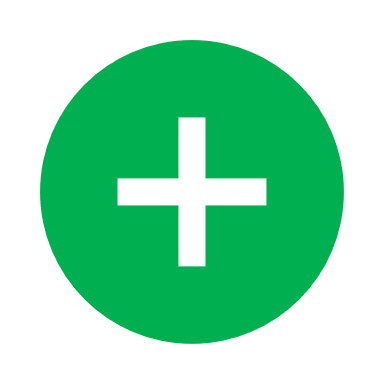 | 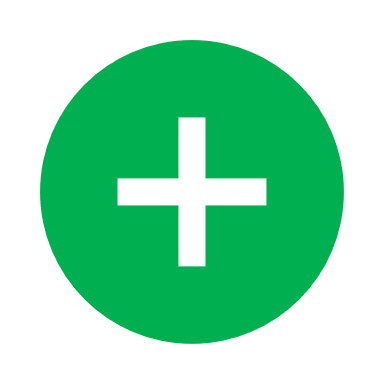 | 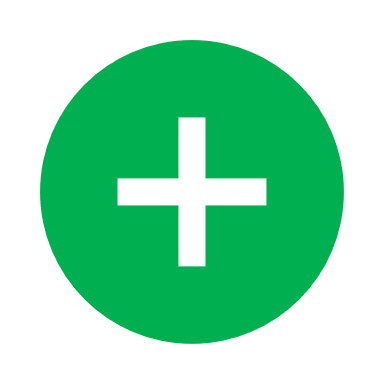 | 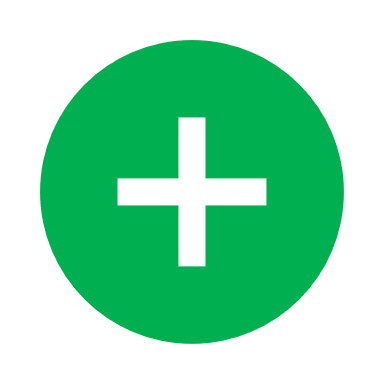 | 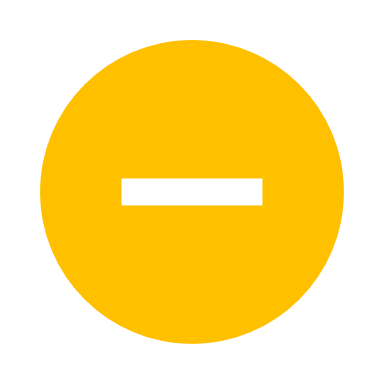 |
| ASCEND (Damjanov)(2) | 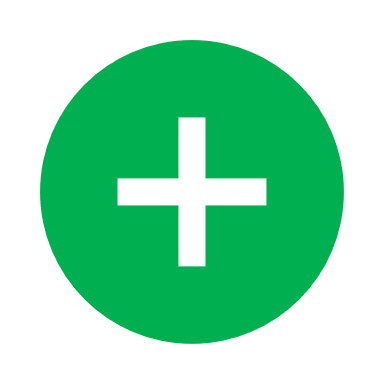 | 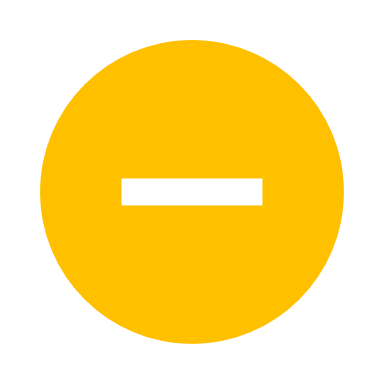 | 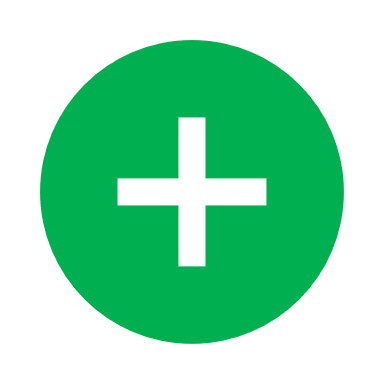 | 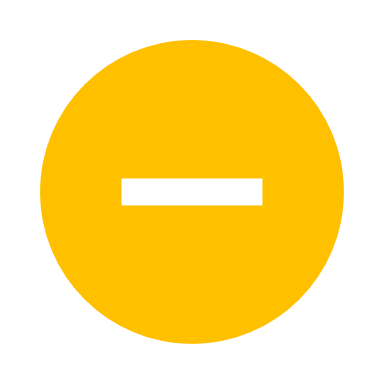 | 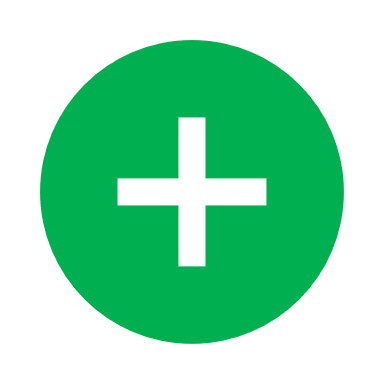 |
| Calin(3) | 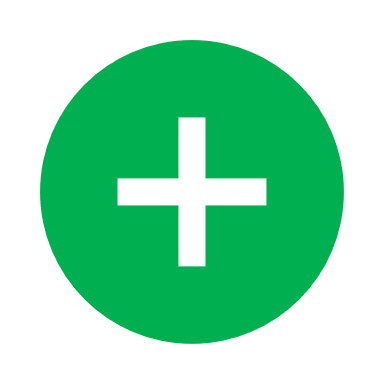 | 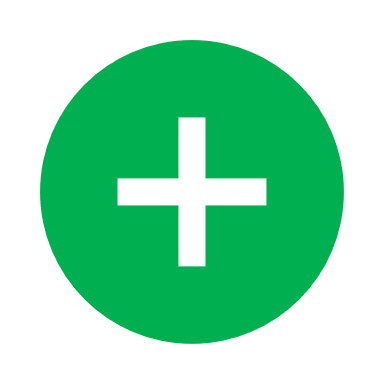 | 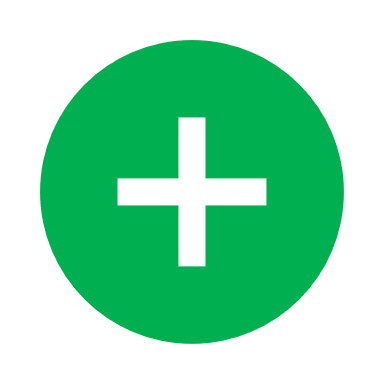 | 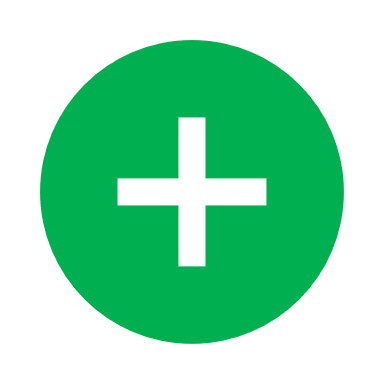 | 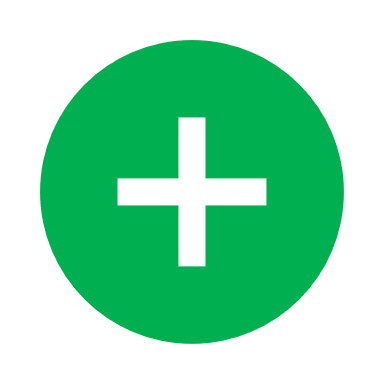 |
| Davis(4) | 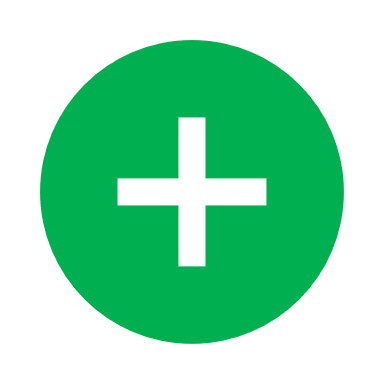 | 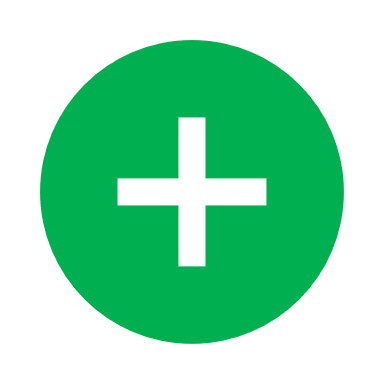 | 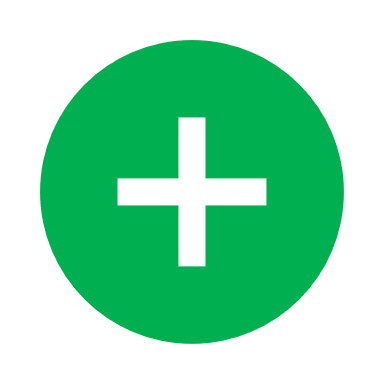 | 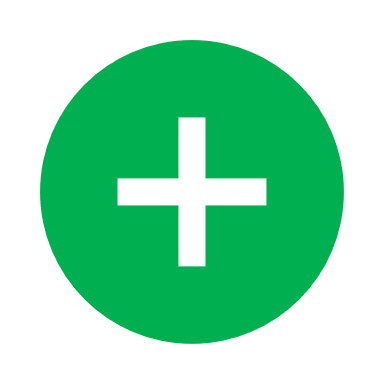 | 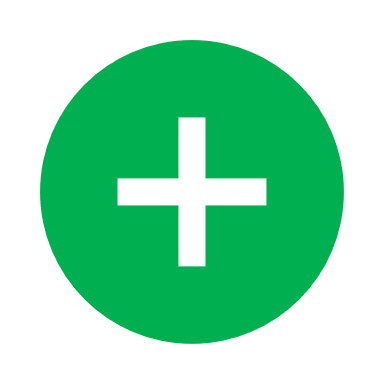 |
| EMBARK (Dougados)(5) | 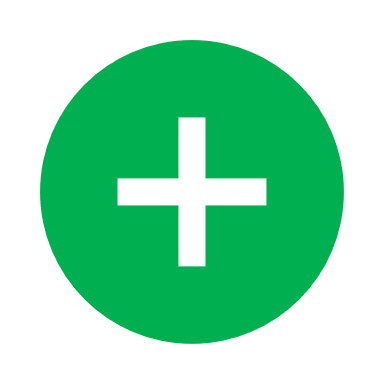 | 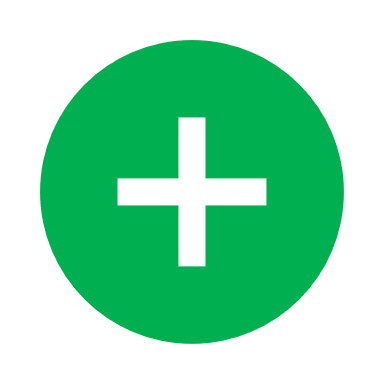 | 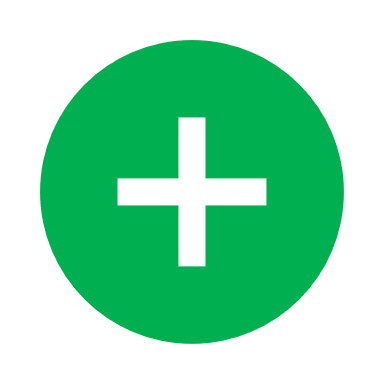 | 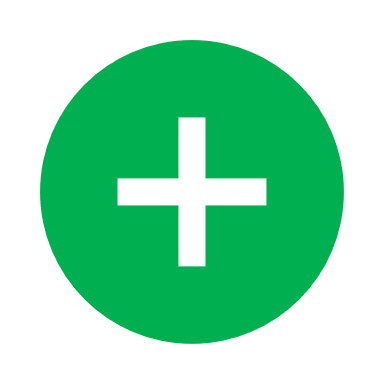 | 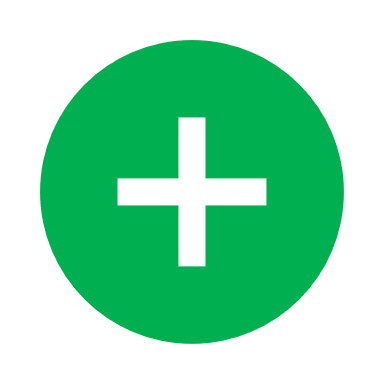 |
| EMBARK (Wei)(6) | 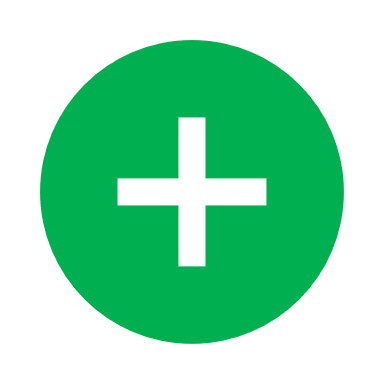 | 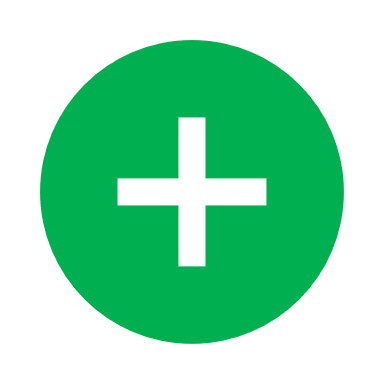 | 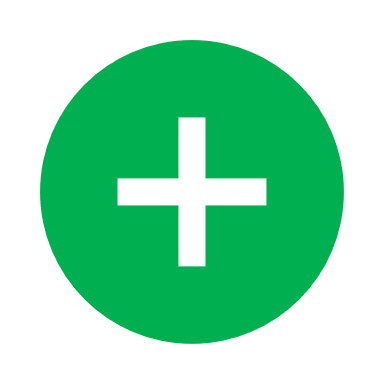 | 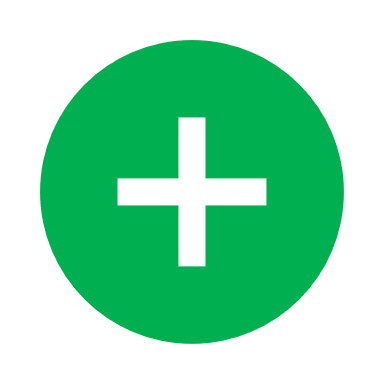 | 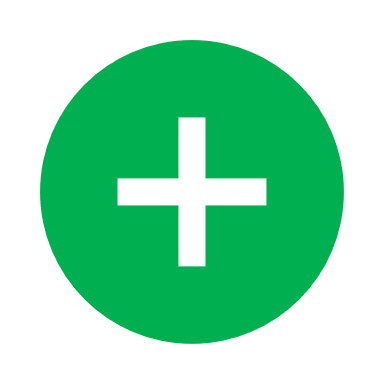 |
| Song(7) | 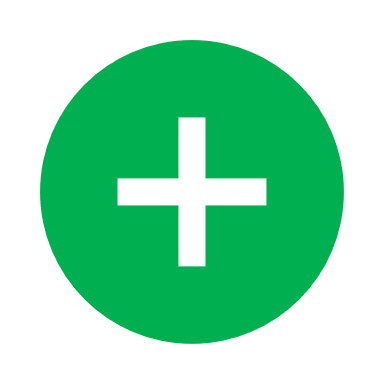 | 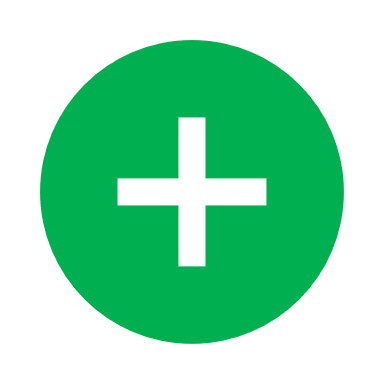 | 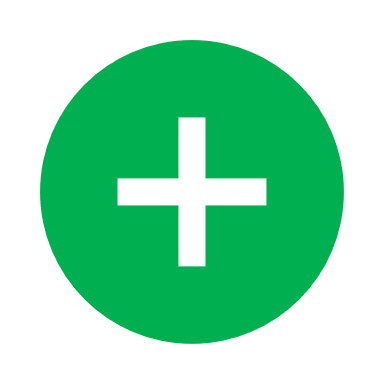 | 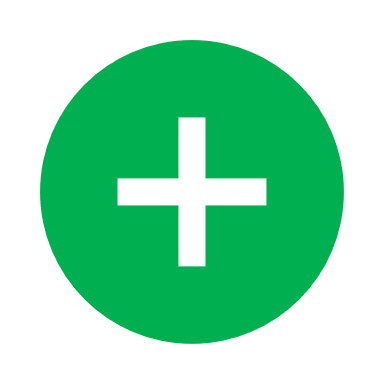 | 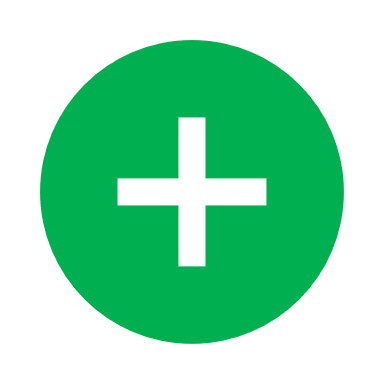 |
| SPARSE (Dougados)(8) | 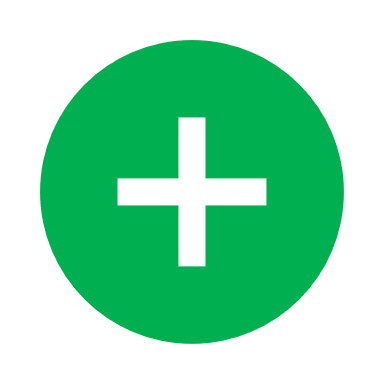 | 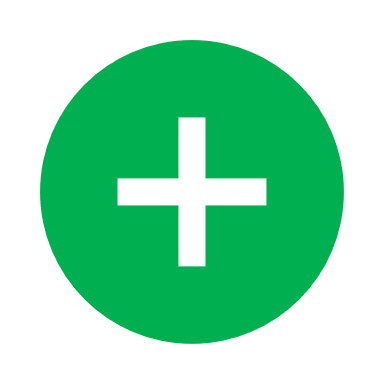 | 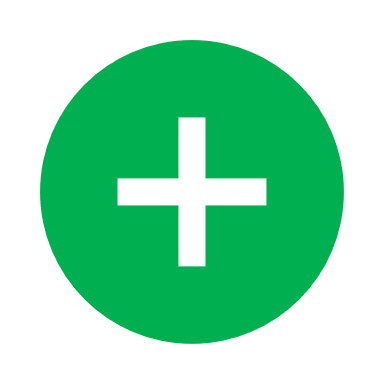 | 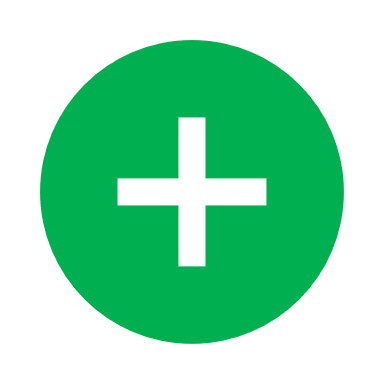 | 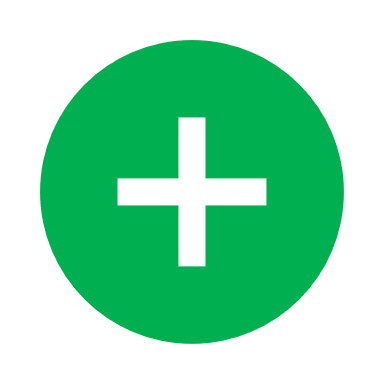 |
| SPINE (Dougados)(9) | 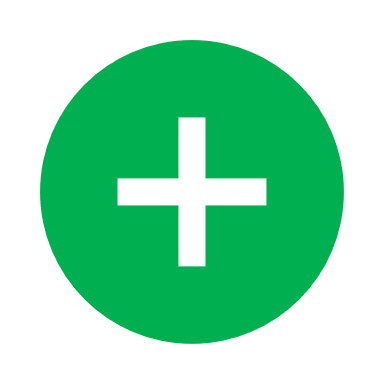 | 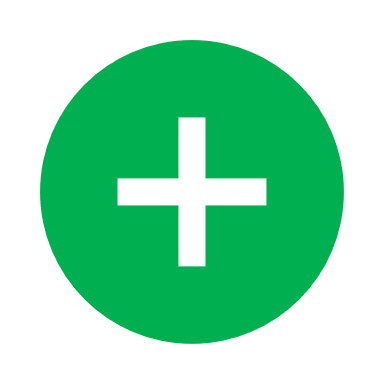 | 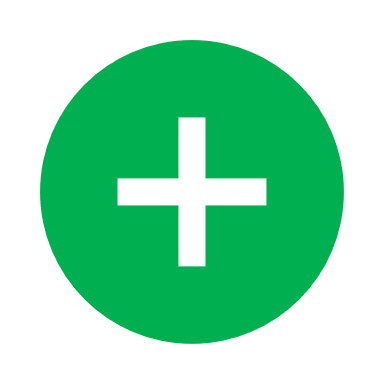 | 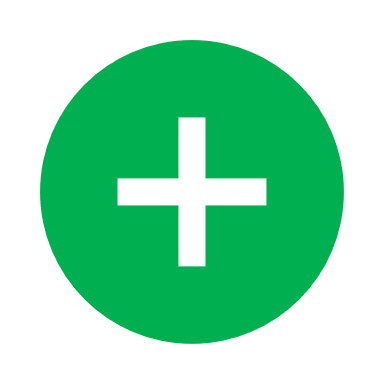 | 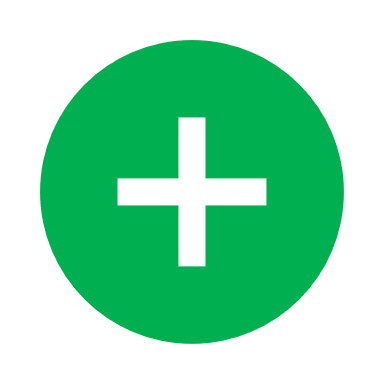 |
| van der Heijde & Silva(10) | 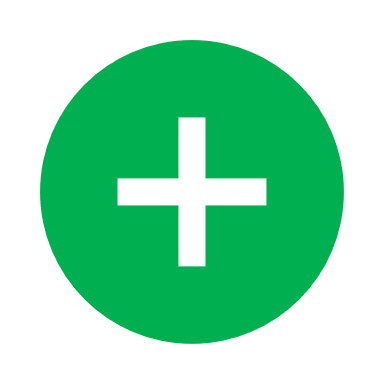 | 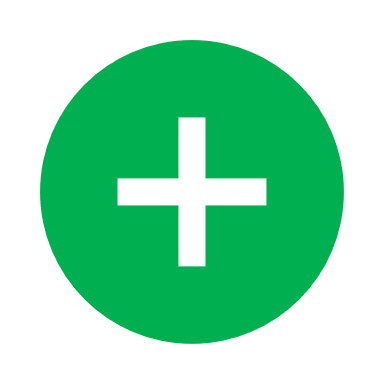 | 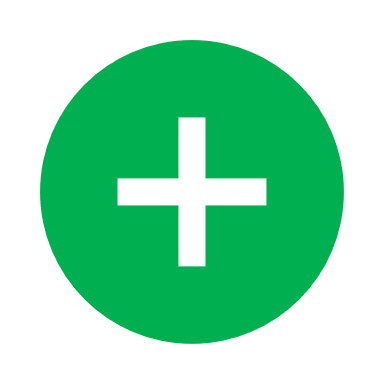 | 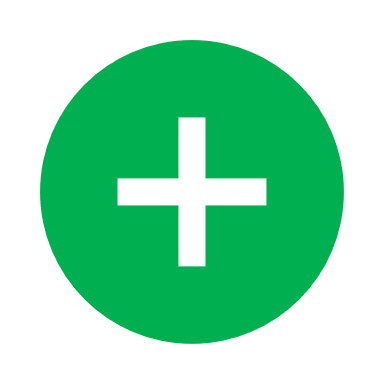 | 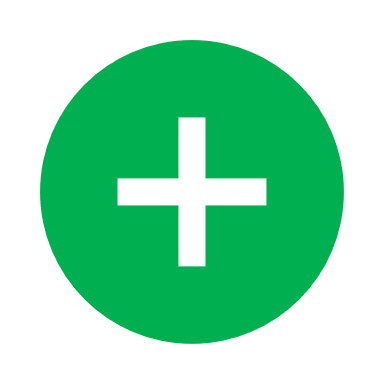 |
| **2. TNFmAb (n = 21)** |  |  |  |  |  |
| ABILITY-1 (Sieper)(11) | 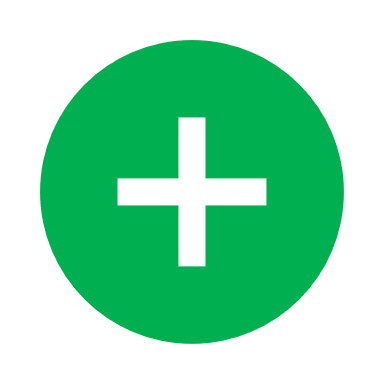 | 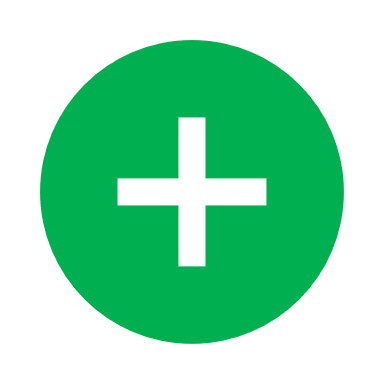 | 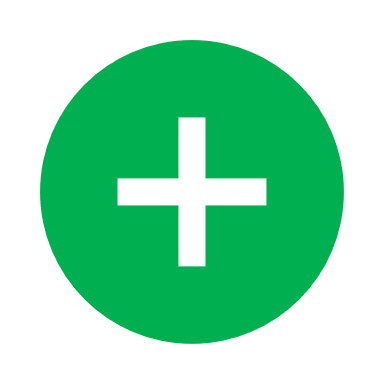 | 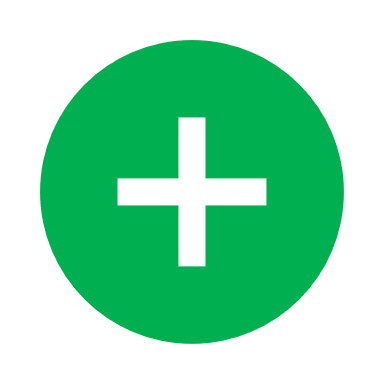 | 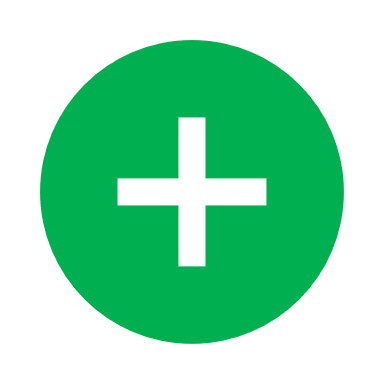 |
| ABILITY-3 (Landewé)(12) | 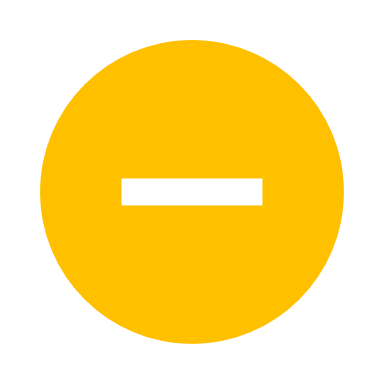 | 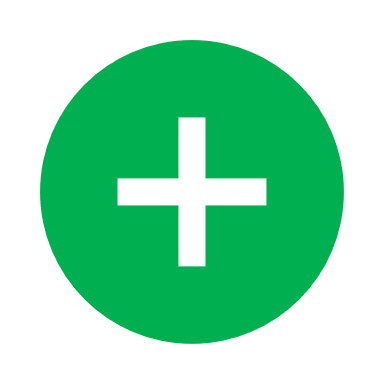 | 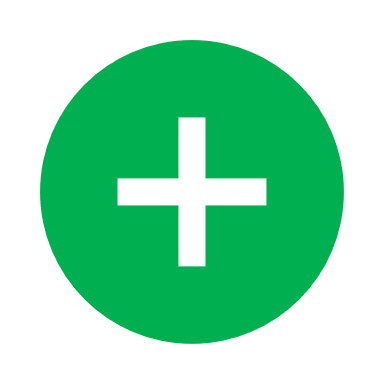 | 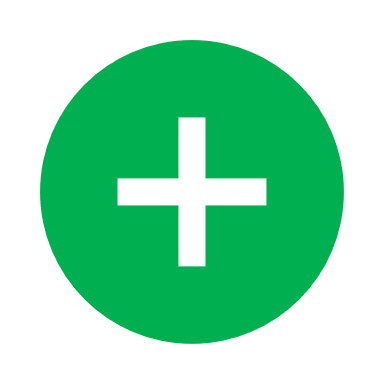 | 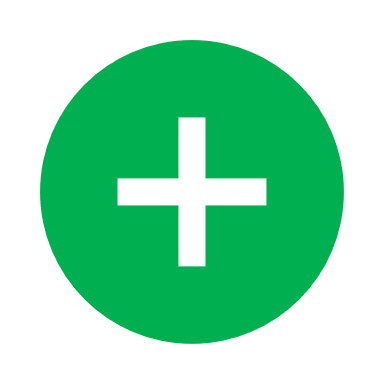 |
| ATLAS (van der Heijde)(13) | 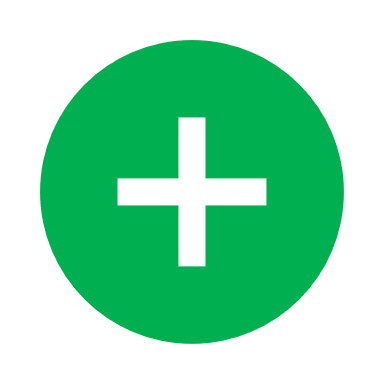 | 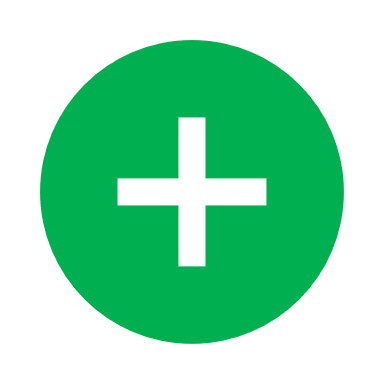 | 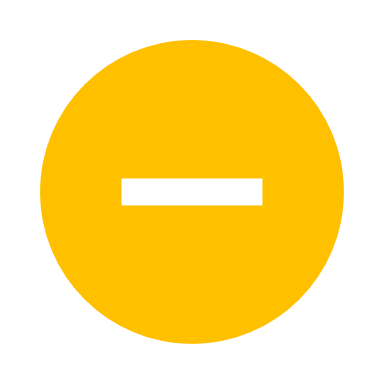 | 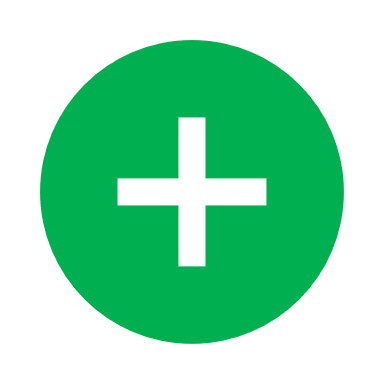 | 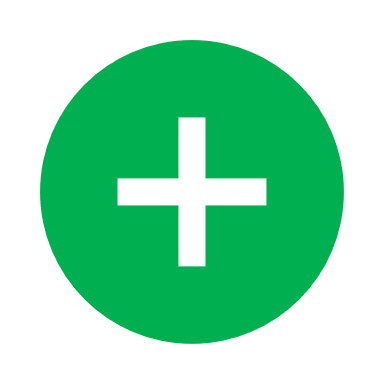 |
| Haibel(14) | 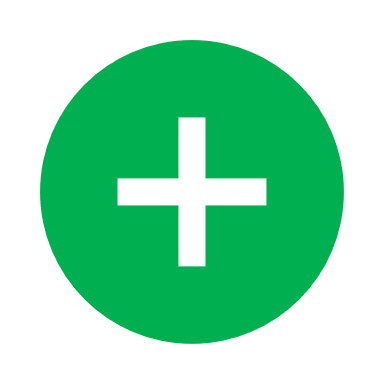 | 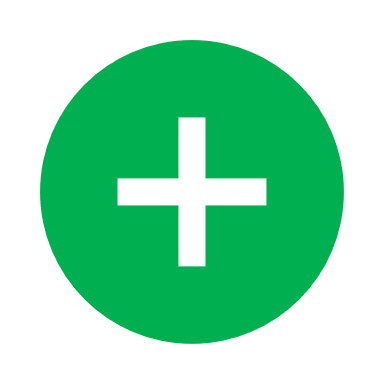 | 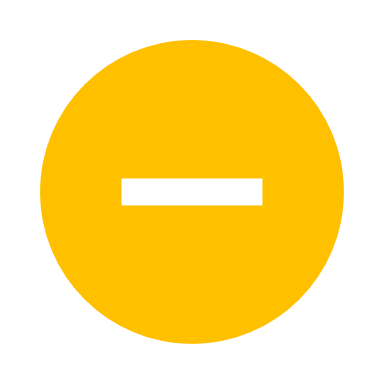 | 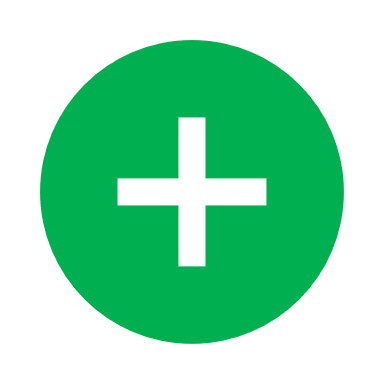 | 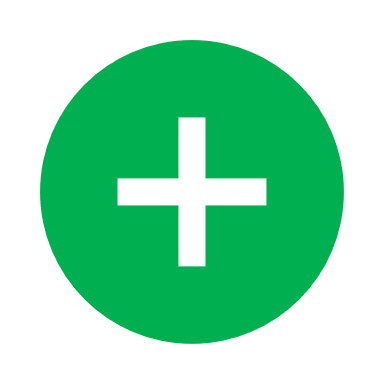 |
| Horneff(15) | 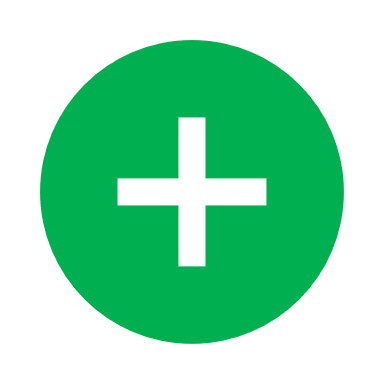 | 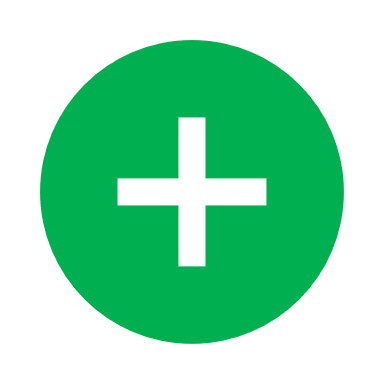 | 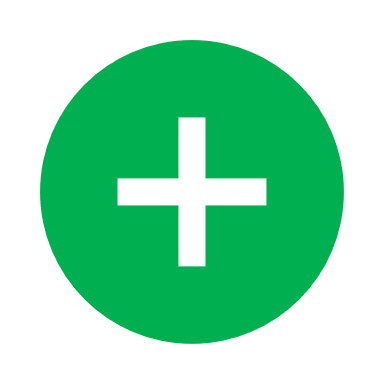 | 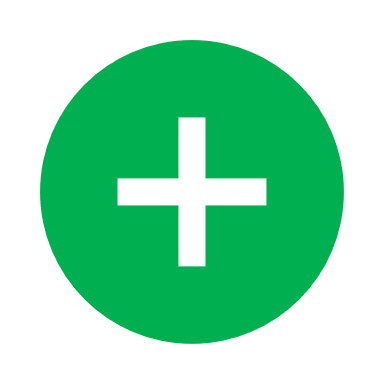 | 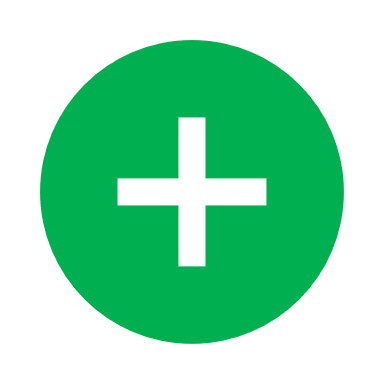 |
| Huang(16) | 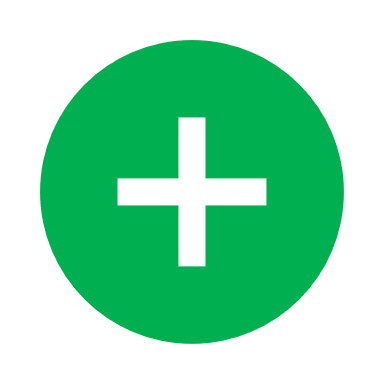 | 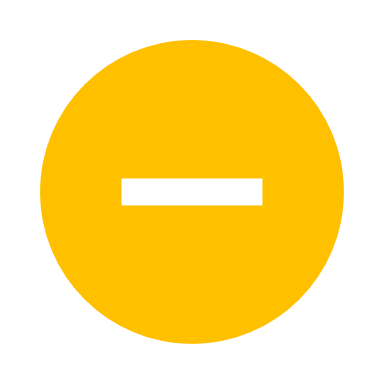 | 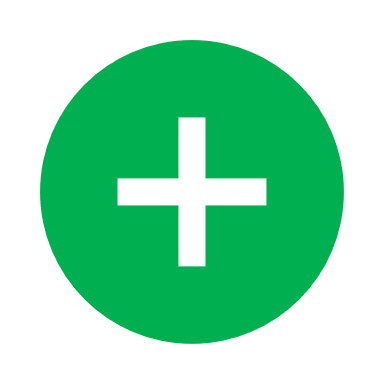 | 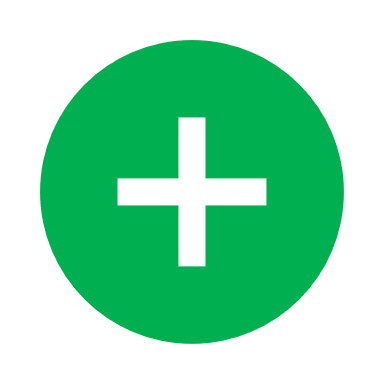 | 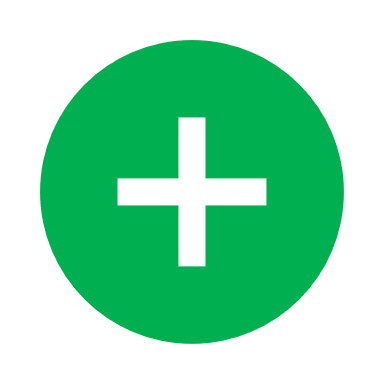 |
| C-axSpAnd (Deodhar)(17) | 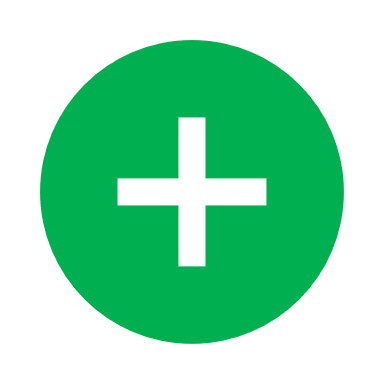 | 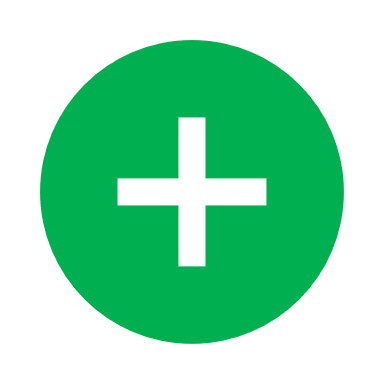 | 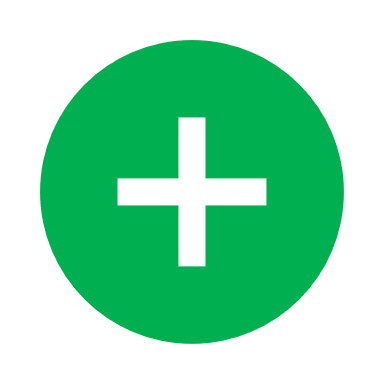 | 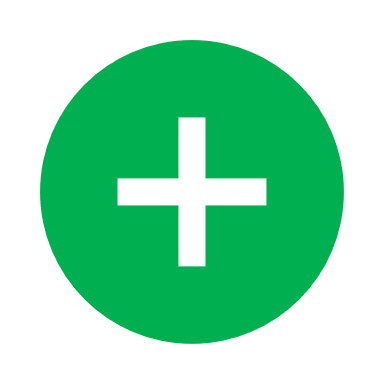 | 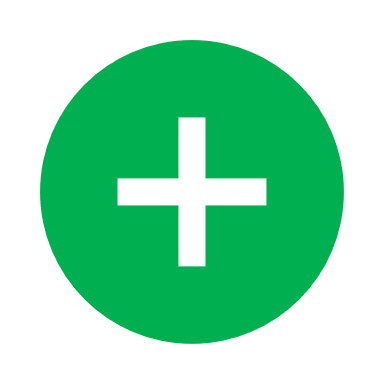 |
| C-OPTIMISE (Landewé)(18) | 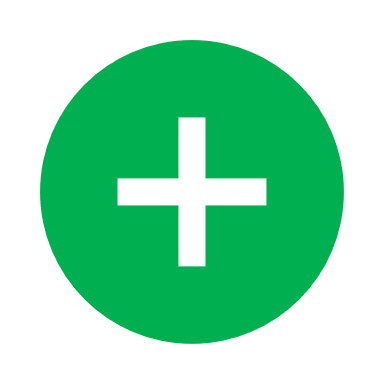 | 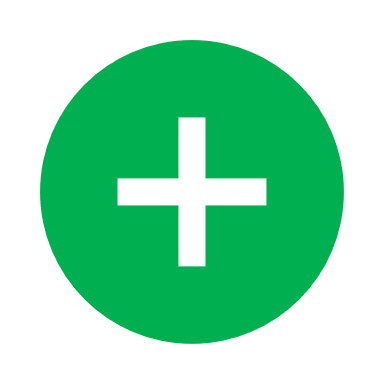 | 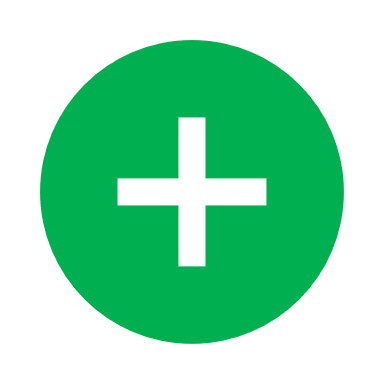 | 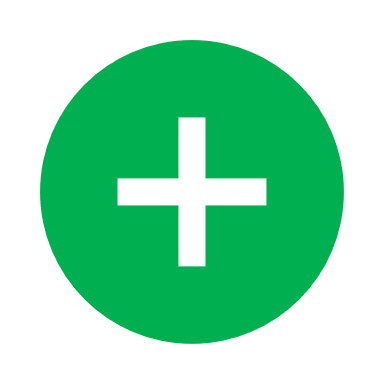 | 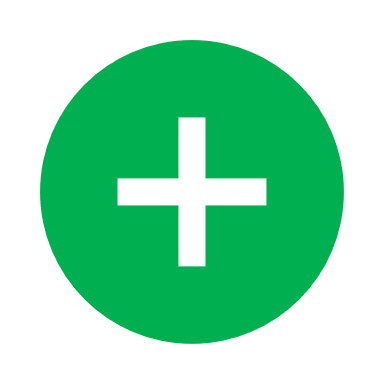 |
| RAPID-axSpA (Landewé)(19) | 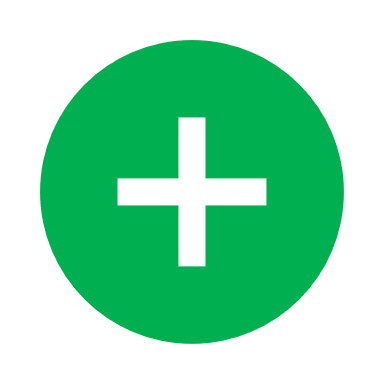 | 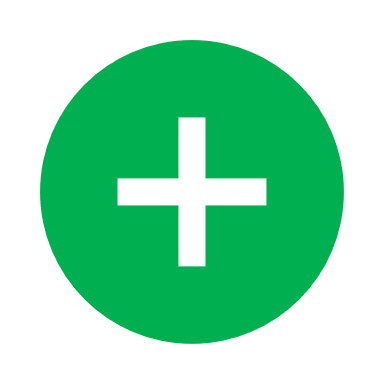 | 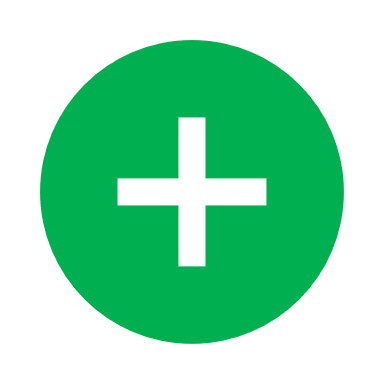 | 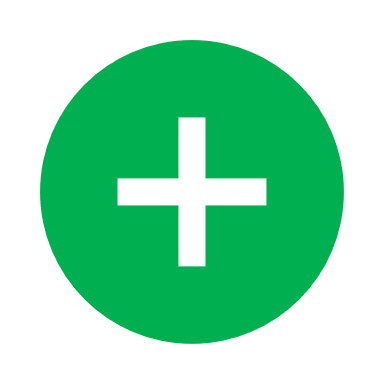 | 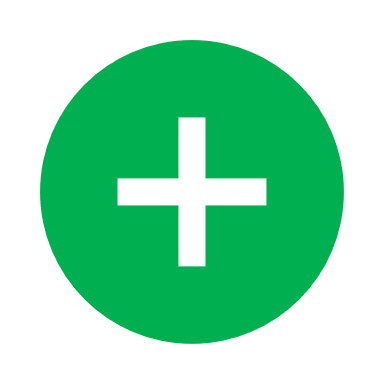 |
| Bao(20) | 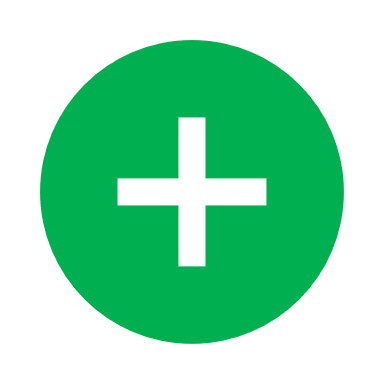 | 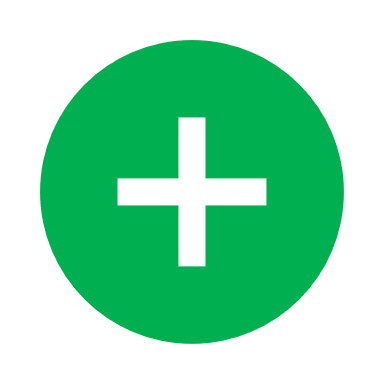 |  |  |  |
| GO-AHEAD (Sieper)(21) |  |  |  |  |  |
| GO-ALIVE (Deodhar)(22) |  |  |  |  |  |
| GO-RAISE (Inman)(23) |  |  |  |  |  |
| Tam(24) |  |  |  |  |  |
| ASSERT (van der Heijde)(25) |  |  |  |  |  |
| Burgos-Vargas(26) |  |  |  |  |  |
| Giardina(27) |  |  |  |  |  |
| INFAST (Sieper)(28) |  |  |  |  |  |
| Inman(29) |  |  |  |  |  |
| Marzo-Ortega(30) |  |  |  |  |  |
| **3. IL17Ai (n = 11)** |  |  |  |  |  |
| COAST-V (van der Heijde)(31) |  |  |  |  |  |
| COAST-W (Deodhar)(32) |  |  |  |  |  |
| COAST-X (Deodhar)(33) |  |  |  |  |  |
| Erdes(34) |  |  |  |  |  |
| Baeten(35) |  |  |  |  |  |
| MEASURE 1 (Baeten)(36) |  |  |  |  |  |
| MEASURE 2 (Baeten)(36) |  |  |  |  |  |
| MEASURE 3 (Pavelka) (37) |  |  |  |  |  |
| MEASURE 4 (Kivitz)(38) |  |  |  |  |  |
| MEASURE 5 (Huang)(39) |  |  |  |  |  |
| PREVENT (Deodhar)(40) |  |  |  |  |  |
| **4.** **IL17A/F/Ri (n = 1)** |  |  |  |  |  |
| BE AGILE (van der Heijde)(41) |  |  |  |  |  |
| **5. IL17RAi (n = 1)** |  |  |  |  |  |
| Wei(42) |  |  |  |  |  |
| **6. JAK1/3i (n = 2)** |  |  |  |  |  |
| Deodhar(43) |  |  |  |  |  |
| van der Heijde(44) |  |  |  |  |  |
| **7. JAK1i (n = 4)** |  |  |  |  |  |
| TORTUGA (van der Heijde)(45) |  |  |  |  |  |
| SELECT-AXIS 1 (van der Heijde)(46) |  |  |  |  |  |
| SELECT-AXIS 2 (AS) (van der Heijde)(47) |  |  |  |  |  |
| SELECT-AXIS 2 (nr-axSpA) (Deodhar)(48) |  |  |  |  |  |
| **8. IL6i (n = 1)** |  |  |  |  |  |
| BUILDER-1 (Sieper)(49) |  |  |  |  |  |
| **9. IL12/23i (n = 2)** |  |  |  |  |  |
| Baeten(50) |  |  |  |  |  |
| Deodhar (study 1)(51) |  |  |  |  |  |
| Deodhar (study 2)(51) |  |  |  |  |  |
| Deodhar (study 3)(51) |  |  |  |  |  |
| **10. PDE4i (n = 2)** |  |  |  |  |  |
| Pathan(52) |  |  |  |  |  |
| Taylor(53) |  |  |  |  |  |
| **11. DMARDs (n = 1)** |  |  |  |  |  |
| Khanna Sharma(54) |  |  |  |  |  |

## Supplementary Table 3. Global inconsistency test (Wald test)

| **Network outcome** | **Chi-square** | **P value** |
| --- | --- | --- |
| ASAS20 | 4.28 | 0.2325 |
| ASAS40 | 7.18 | 0.0663 |
| BASDAI50 | 11.78 | 0.0082 |
| ASDAS-ID | 1.51 | 0.4702 |
| TEAE | 0.64 | 0.7259 |
| SAE | 2.39 | 0.4962 |

## Supplementary Table 4. Local inconsistency test (node splitting)

|  | **Trial comparisons** | Direct | | Indirect | | Difference | | P **value** | Tau |
| --- | --- | --- | --- | --- | --- | --- | --- | --- | --- |
|  |  | Coef. | Std. Err. | Coef. | Std. Err. | Coef. | Std. Err. |  |  |
| ASAS20 | TNFRFc vs. TNFmAb | 0.75 | 0.71 | 0.22 | 0.22 | 0.52 | 0.74 | 0.48 | 0.34 |
|  | TNFRFc vs. PLA | -1.13 | 0.19 | -0.61 | 0.72 | -0.52 | 0.74 | 0.48 | 0.34 |
|  | TNFmAb vs. IL17Ai | 0.31 | 0.41 | -0.54 | 0.18 | 0.85 | 0.45 | 0.06 | 0.31 |
|  | TNFmAb vs. PLA | -1.38 | 0.12 | -1.20 | 0.54 | -0.18 | 0.56 | 0.75 | 0.34 |
|  | IL17Ai vs. PLA* | -0.92 | 0.14 | -2.45 | 0.77 | 1.54 | 0.78 | 0.05 | 0.31 |
|  | IL17A/Fi vs. PLA* | -1.27 | 0.46 | -1.41 | 1270.74 | 0.14 | 1270.74 | 1.00 | 0.33 |
|  | IL17RAi vs. PLA* | -1.06 | 0.47 | -2.20 | 1268.14 | 1.14 | 1268.14 | 1.00 | 0.33 |
|  | JAK1/3i vs. PLA* | -1.05 | 0.31 | -1.96 | 914.30 | 0.92 | 914.30 | 1.00 | 0.33 |
|  | JAK1i vs. PLA* | -1.11 | 0.22 | -2.24 | 629.24 | 1.13 | 629.24 | 1.00 | 0.33 |
|  | IL6i vs. PLA* | -0.45 | 0.54 | -2.20 | 1356.06 | 1.75 | 1356.06 | 1.00 | 0.33 |
|  | IL12/23i vs. PLA* | -0.39 | 0.23 | -2.05 | 663.87 | 1.66 | 663.87 | 1.00 | 0.33 |
|  | PDE4i vs. PLA* | -0.30 | 0.36 | -2.19 | 1005.20 | 1.89 | 1005.20 | 1.00 | 0.33 |
| ASAS40 | TNFRFc vs. TNFmAb | 0.48 | 0.67 | 0.44 | 0.28 | 0.04 | 0.72 | 0.95 | 0.35 |
|  | TNFRFc vs. PLA | -1.08 | 0.25 | -1.04 | 0.68 | -0.04 | 0.72 | 0.95 | 0.35 |
|  | TNFmAb vs. IL17Ai | 0.59 | 0.40 | -0.60 | 0.18 | 1.19 | 0.44 | 0.01 | 0.29 |
|  | TNFmAb vs. PLA | -1.56 | 0.12 | -0.82 | 0.53 | -0.74 | 0.55 | 0.18 | 0.33 |
|  | IL17Ai vs. PLA* | -1.05 | 0.14 | -3.02 | 0.78 | 1.97 | 0.80 | 0.01 | 0.30 |
|  | IL17A/Fi vs. PLA* | -1.51 | 0.53 | -1.11 | 1321.27 | -0.41 | 1321.27 | 1.00 | 0.35 |
|  | IL17RAi vs. PLA* | -0.90 | 0.49 | -2.09 | 1398.89 | 1.19 | 1398.89 | 1.00 | 0.35 |
|  | JAK1/3i vs. PLA* | -1.36 | 0.35 | -1.66 | 963.97 | 0.30 | 963.97 | 1.00 | 0.35 |
|  | JAK1i vs. PLA* | -1.13 | 0.23 | -2.05 | 683.18 | 0.92 | 683.18 | 1.00 | 0.35 |
|  | IL6i vs. PLA* | 0.60 | 0.66 | -2.09 | 1743.73 | 2.70 | 1743.73 | 1.00 | 0.35 |
|  | IL12/23i vs. PLA* | -0.28 | 0.25 | -2.04 | 767.16 | 1.76 | 767.16 | 1.00 | 0.35 |
|  | PDE4i vs. PLA* | -0.12 | 0.41 | -2.32 | 1176.76 | 2.21 | 1176.76 | 1.00 | 0.35 |
| BASDAI50 | TNFRFc vs. SSZ | -0.97 | 0.47 | 1.84 | 0.86 | -2.81 | 0.98 | 0.00 | 0.40 |
|  | TNFRFc vs. PLA | -0.92 | 0.29 | -3.73 | 0.94 | 2.81 | 0.98 | 0.00 | 0.40 |
|  | TNFmAb vs. IL17Ai | 0.45 | 0.52 | -0.69 | 0.48 | 1.14 | 0.71 | 0.11 | 0.44 |
|  | TNFmAb vs. PLA* | -1.42 | 0.16 | 0.15 | 1.38 | -1.57 | 1.39 | 0.26 | 0.46 |
|  | IL17Ai vs. PLA* | -1.02 | 0.37 | -2.61 | 1.00 | 1.60 | 1.09 | 0.14 | 0.45 |
|  | JAK1/3i vs. PLA* | -0.87 | 0.60 | -1.85 | 1313.15 | 0.99 | 1313.15 | 1.00 | 0.47 |
|  | JAK1i vs. PLA* | -1.09 | 0.31 | -2.24 | 784.24 | 1.15 | 784.24 | 1.00 | 0.47 |
|  | IL12/23i vs. PLA* | -0.28 | 0.33 | -2.25 | 829.26 | 1.97 | 829.26 | 1.00 | 0.47 |
|  | SSZ vs. PLA | -2.76 | 0.81 | 0.05 | 0.55 | -2.81 | 0.98 | 0.00 | 0.40 |
| ASDAS-ID | TNFRFc vs. PLA* | -0.99 | 0.38 | -1.12 | 220.17 | 0.14 | 220.18 | 1.00 | 0.49 |
|  | TNFmAb vs. IL17Ai | -0.16 | 0.61 | -1.05 | 0.60 | 0.88 | 0.86 | 0.31 | 0.49 |
|  | TNFmAb vs. PLA* | -1.92 | 0.25 | 0.09 | 1.58 | -2.01 | 1.62 | 0.21 | 0.48 |
|  | IL17Ai vs. PLA* | -1.09 | 0.49 | -2.05 | 1.32 | 0.96 | 1.50 | 0.52 | 0.50 |
|  | JAK1/3i vs. PLA* | -0.66 | 0.75 | -1.58 | 1849.93 | 0.93 | 1849.93 | 1.00 | 0.49 |
|  | JAK1i vs. PLA* | -1.73 | 0.45 | -1.44 | 1284.15 | -0.29 | 1284.15 | 1.00 | 0.49 |
|  | IL12/23i vs. PLA* | -0.37 | 0.50 | -1.82 | 1723.30 | 1.45 | 1723.30 | 1.00 | 0.49 |
| TEAE | TNFRFc vs. PLA* | -0.40 | 0.17 | -0.12 | 103.16 | -0.29 | 103.16 | 1.00 | 0.00 |
|  | TNFmAb vs. C | -0.26 | 0.26 | -0.04 | 0.11 | -0.22 | 0.28 | 0.44 | 0.00 |
|  | TNFmAb vs. PLA* | -0.35 | 0.07 | -0.73 | 0.47 | 0.37 | 0.47 | 0.43 | 0.00 |
|  | IL17Ai vs. PLA* | -0.30 | 0.08 | -0.01 | 0.52 | -0.29 | 0.53 | 0.58 | 0.00 |
|  | IL17A/Fi vs. PLA* | 0.23 | 0.29 | -1.06 | 1366.98 | 1.29 | 1366.98 | 1.00 | 0.00 |
|  | IL17RAi vs. PLA* | 0.08 | 0.32 | -0.84 | 1270.37 | 0.92 | 1270.37 | 1.00 | 0.00 |
|  | JAK1/3i vs. PLA* | -0.19 | 0.20 | -0.83 | 799.19 | 0.64 | 799.19 | 1.00 | 0.00 |
|  | JAK1i vs. PLA* | -0.15 | 0.13 | -1.10 | 672.77 | 0.95 | 672.77 | 1.00 | 0.00 |
|  | IL6i vs. PLA* | -0.63 | 0.43 | -0.77 | 1304.62 | 0.14 | 1304.62 | 1.00 | 0.00 |
|  | IL12/23i vs. PLA* | 0.06 | 0.13 | -0.74 | 567.71 | 0.80 | 567.71 | 1.00 | 0.00 |
|  | PDE4i vs. PLA* | -0.15 | 0.19 | -0.25 | 1074.63 | 0.10 | 1074.63 | 1.00 | 0.00 |
| SAE | TNFRFc vs. TNFmAb | -0.74 | 1.26 | -0.08 | 0.44 | -0.65 | 1.33 | 0.62 | 0.00 |
|  | TNFRFc vs. PLA | -0.05 | 0.38 | -0.70 | 1.28 | 0.65 | 1.33 | 0.62 | 0.00 |
|  | TNFmAb vs. IL17Ai | -1.00 | 0.84 | -0.11 | 0.32 | -0.89 | 0.90 | 0.32 | 0.00 |
|  | TNFmAb vs. PLA | 0.05 | 0.22 | 0.06 | 1.04 | -0.01 | 1.08 | 0.99 | 0.00 |
|  | IL17Ai vs. PLA* | 0.21 | 0.23 | 2.35 | 1.66 | -2.14 | 1.70 | 0.21 | 0.00 |
|  | IL17A/Fi vs. PLA* | 1.02 | 0.93 | -0.73 | 4739.40 | 1.74 | 4739.40 | 1.00 | 0.00 |
|  | IL17RAi vs. PLA* | -1.41 | 1.13 | 0.23 | 3593.92 | -1.65 | 3593.92 | 1.00 | 0.00 |
|  | JAK1/3i vs. PLA* | 0.39 | 0.78 | -0.50 | 3620.52 | 0.89 | 3620.52 | 1.00 | 0.00 |
|  | JAK1i vs. PLA* | -0.96 | 0.58 | -0.07 | 2456.99 | -0.89 | 2456.99 | 1.00 | 0.00 |
|  | IL6i vs. PLA* | -1.65 | 1.56 | 0.38 | 3718.58 | -2.03 | 3718.58 | 1.00 | 0.00 |
|  | IL12/23i vs. PLA* | -0.02 | 0.51 | -0.30 | 2227.62 | 0.29 | 2227.62 | 1.00 | 0.00 |
|  | PDE4i vs. PLA* | -1.20 | 0.94 | 0.08 | 3034.61 | -1.28 | 3034.61 | 1.00 | 0.00 |

## Supplementary Figure 1. League plot for BASDAI50 and ASDAS-ID

| **TNFRFc** | 0.42 (0.17-1.03) | 0.77 (0.26-2.33) | 1.39 (0.27-7.19) | 0.48 (0.15-1.51) | 1.86 (0.54-6.37) | 4.08 (1.18-14.08) | 2.68 (1.28-5.63) |
| --- | --- | --- | --- | --- | --- | --- | --- |
| 0.80 (0.41-1.55) | **TNFmAb** | 1.84 (0.80-4.25) | 3.31 (0.70-15.51) | 1.13 (0.42-3.05) | 4.42 (1.48-13.25) | 9.71 (2.11-44.69) | 6.38 (3.92-10.38) |
| 0.94 (0.38-2.33) | 1.18 (0.58-2.43) | **IL17Ai** | 1.80 (0.34-9.58) | 0.61 (0.19-2.00) | 2.40 (0.67-8.57) | 5.28 (1.01-27.64) | 3.46 (1.55-7.77) |
| 1.35 (0.36-5.03) | 1.70 (0.51-5.73) | 1.44 (0.37-5.61) | **JAK1/3i** | 0.34 (0.06-1.89) | 1.34 (0.23-7.82) | 2.94 (0.38-23.00) | 1.93 (0.44-8.36) |
| 1.08 (0.46-2.53) | 1.36 (0.68-2.71) | 1.15 (0.46-2.90) | 0.80 (0.21-3.00) | **JAK1i** | 3.91 (1.05-14.64) | 8.59 (1.58-46.73) | 5.64 (2.34-13.60) |
| 2.45 (1.02-5.88) | 3.07 (1.49-6.34) | 2.60 (1.01-6.71) | 1.81 (0.47-6.90) | 2.26 (0.92-5.53) | **IL12/23i** | 2.19 (0.38-12.58) | 1.44 (0.54-3.85) |
| 1.27 (0.50-3.23) | 1.60 (0.55-4.63) | 1.35 (0.40-4.61) | 0.94 (0.20-4.43) | 1.17 (0.36-3.85) | 0.52 (0.16-1.73) | **SSZ** | 0.66 (0.16-2.78) |
| 3.23 (1.79-5.82) | 4.05 (2.96-5.56) | 3.43 (1.72-6.84) | 2.38 (0.74-7.70) | 2.98 (1.61-5.51) | 1.32 (0.69-2.53) | 2.54 (0.92-7.03) | **PLA** |

▆ BASDAI50 ▆ Comparison ▆ ASDAS-ID

## Supplementary Figure 2. Forest plots (Predictive interval plot) for all outcomes

The parameters of 95%PrIs are crucial in effectively appraising heterogeneity among the included studies and interpreting results of the future trials by giving the range within which the results of a future study might lie.(55, 56) However, this was frequently absent in the previous network meta-analysis. In our study, according to the mean effect and its respective 95%PrIs, better efficacy for ASAS20 response was identified in the comparisons of treatments including TNFRFc vs. (SSZ or PLA), TNFmAb vs. (IL12/23i, PDE4i, SSZ, or PLA), (IL17Ai, JAK1/3i, or JAK1i) vs. PLA, and JAK1i vs. SSZ. Concurrently, these data suggested a low heterogeneity, which indicating that future head-to-head trials amongst these comparisons of biologics may have similar probabilities of response rate as the results conducted in our network meta-analysis.

In these plots, the diamonds represent the mean of summary odds ratios (ORs) for each comparison; the black lines represent the 95% confidence intervals of ORs (95%CI); the red lines indicate respective 95% Predictive Intervals (PrI), which provide an interval within which the estimate of a future study is expected to be. The blue vertical line is the line of no effect (OR equal to 1).

**ASAS20 ASAS40**

**BASDAI50 ASDAS-ID**

**TEAEs SAEs**

## Supplementary Figure 3. SUCRA plot for efficacy and safety outcomes. SUCRA is frequently used to estimate the probability of each relative treatment will be effective. A larger SUCRA score indicate a higher ranking, which suggests a higher probability of efficacy.(57)

## Supplementary Figure 4. Evidence plots for subgroup analysis

## Supplementary Figure 5. League plot for ASAS20 and ASAS40 in ankylosing spondylitis (AS)

| **TNFRFc** | 0.75  (0.39-1.44) | 0.98  (0.50-1.94) | 0.75  (0.24-2.37) | 1.50  (0.48-4.68) | 0.87  (0.36-2.11) | 1.06  (0.48-2.31) | 6.24  (1.57-24.84) | 2.58  (1.15-5.81) | 3.13  (1.21-8.10) | 3.25  (1.95-5.40) | 3.41  (1.84-6.32) |
| --- | --- | --- | --- | --- | --- | --- | --- | --- | --- | --- | --- |
| 1.03  (0.68-1.56) | **TNFmAb** | 1.31  (0.88-1.94) | 1.00  (0.36-2.77) | 2.00  (0.73-5.45) | 1.17  (0.58-2.34) | 1.41  (0.80-2.49) | 8.34  (2.34-29.72) | 3.45  (1.89-6.29) | 4.18  (1.92-9.09) | 4.34  (1.90-9.93) | 4.56  (3.41-6.11) |
| 1.47  (0.96-2.25) | 1.43  (1.07-1.91) | **IL17Ai** | 0.77  (0.28-2.12) | 1.53  (0.56-4.17) | 0.89  (0.44-1.79) | 1.08  (0.61-1.91) | 6.37  (1.79-22.73) | 2.64  (1.44-4.82) | 3.19  (1.46-6.97) | 3.31  (1.42-7.76) | 3.48  (2.59-4.69) |
| 1.15  (0.52-2.52) | 1.12  (0.54-2.31) | 0.78  (0.38-1.62) | **IL17A/Fi** | 1.99  (0.51-7.81) | 1.16  (0.37-3.71) | 1.41  (0.48-4.17) | 8.31  (1.72-40.08) | 3.44  (1.14-10.39) | 4.17  (1.24-14.00) | 4.33  (1.23-15.23) | 4.55  (1.72-12.02) |
| N/A | N/A | N/A | N/A | **IL17RAi** | 0.58  (0.19-1.84) | 0.71  (0.24-2.07) | 4.17  (0.87-19.95) | 1.73  (0.58-5.15) | 2.09  (0.63-6.95) | 2.17  (0.62-7.57) | 2.28  (0.87-5.95) |
| 1.42  (0.79-2.57) | 1.39  (0.83-2.30) | 0.97  (0.58-1.62) | 1.24  (0.54-2.86) | N/A | **JAK1/3i** | 1.21  (0.55-2.67) | 7.14  (1.78-28.60) | 2.95  (1.30-6.71) | 3.58  (1.37-9.35) | 3.72  (1.34-10.27) | 3.90  (2.08-7.33) |
| 1.28  (0.76-2.16) | 1.25  (0.81-1.91) | 0.87  (0.57-1.34) | 1.12  (0.51-2.45) | N/A | 0.90  (0.50-1.62) | **JAK1i** | 5.90  (1.56-22.26) | 2.44  (1.19-4.99) | 2.96  (1.24-7.08) | 3.07  (1.21-7.81) | 3.23  (1.99-5.23) |
| 2.60  (0.99-6.85) | 2.53  (1.01-6.36) | 1.77  (0.71-4.46) | 2.27  (0.73-7.05) | N/A | 1.83  (0.67-5.00) | 2.03  (0.77-5.35) | **IL6i** | 0.41  (0.11-1.59) | 0.50  (0.12-2.10) | 0.52  (0.12-2.27) | 0.55  (0.16-1.88) |
| 2.65  (1.54-4.57) | 2.58  (1.64-4.06) | 1.81  (1.16-2.83) | 2.31  (1.04-5.15) | N/A | 1.86  (1.01-3.42) | 2.07  (1.21-3.55) | 1.02  (0.38-2.72) | **IL12/23i** | 1.21  (0.50-2.95) | 1.26  (0.48-3.28) | 1.32  (0.78-2.24) |
| 3.24  (1.74-6.01) | 3.15  (1.84-5.40) | 2.21  (1.29-3.77) | 2.82  (1.20-6.62) | N/A | 2.27  (1.16-4.47) | 2.53  (1.37-4.67) | 1.25  (0.45-3.46) | 1.22  (0.65-2.29) | **PDE4i** | 1.04  (0.35-3.06) | 1.09  (0.53-2.25) |
| 2.61  (1.77-3.84) | 2.54  (1.44-4.48) | 1.78  (1.00-3.16) | 2.27  (0.95-5.46) | N/A | 1.83  (0.90-3.70) | 2.03  (1.06-3.89) | 1.00  (0.35-2.84) | 0.98  (0.50-1.91) | 0.80  (0.39-1.67) | **SSZ** | 1.05  (0.47-2.34) |
| 4.08  (2.82-5.90) | 3.97  (3.20-4.92) | 2.78  (2.24-3.45) | 3.56  (1.77-7.13) | N/A | 2.87  (1.81-4.54) | 3.18  (2.20-4.60) | 1.57  (0.64-3.85) | 1.54  (1.03-2.29) | 1.26  (0.77-2.06) | 1.57  (0.92-2.67) | **PLA** |

▆ ASAS20 ▆ Comparison ▆ ASAS40 N/A, not available

## Supplementary Figure 6. League plot for ASAS20 and ASAS40 in non-radiographic axial Spondyloarthritis (nr-axSpA)

| **TNFRFc** | 0.60  (0.27-1.34) | 1.19  (0.49-2.89) | 1.23  (0.19-7.93) | 0.87  (0.30-2.47) | 1.88  (0.63-5.67) | 2.45  (1.22-4.94) |
| --- | --- | --- | --- | --- | --- | --- |
| 0.65  (0.38-1.09) | **TNFmAb** | 1.99  (1.02-3.89) | 2.05  (0.35-12.12) | 1.45  (0.60-3.49) | 3.16  (1.23-8.08) | 4.11  (2.75-6.14) |
| 1.15  (0.65-2.03) | 1.78  (1.13-2.80) | **IL17Ai** | 1.03  (0.17-6.33) | 0.73  (0.28-1.89) | 1.59  (0.58-4.36) | 2.07  (1.20-3.57) |
| N/A | N/A | N/A | **IL17RAi** | 0.70  (0.11-4.70) | 1.54  (0.22-10.55) | 2.00  (0.35-11.27) |
| 0.72  (0.38-1.37) | 1.12  (0.66-1.92) | 0.63  (0.35-1.12) | N/A | **JAK1i** | 2.18  (0.69-6.92) | 2.84  (1.30-6.20) |
| 1.52  (0.77-3.03) | 2.36  (1.30-4.29) | 1.32  (0.70-2.50) | N/A | 2.10  (1.04-4.23) | **IL12/23i** | 1.30  (0.56-3.05) |
| 1.85  (1.19-2.87) | 2.86  (2.16-3.78) | 1.60  (1.13-2.29) | N/A | 2.55  (1.61-4.03) | 1.21  (0.71-2.06) | **PLA** |

▆ ASAS20 ▆ Comparison ▆ ASAS40 N/A, not available

## Supplementary Figure 7. Cluster SUCRA ranking plot for ankylosing spondylitis (AS) and axial spondyloarthritis (nr-axSpA)

## Supplementary Figure 8. Funnel plot

**ASAS20**

**ASAS40**

**BASDAI50**

**ASDAS-ID**

**TEAEs**

**SAEs**

## Supplementary References.

1. Braun J, van der Horst-Bruinsma IE, Huang F, Burgos-Vargas R, Vlahos B, Koenig AS, et al. Clinical efficacy and safety of etanercept versus sulfasalazine in patients with ankylosing spondylitis: a randomized, double-blind trial. Arthritis Rheum. 2011;63(6):1543-51.

2. Damjanov N, Shehhi WA, Huang F, Kotak S, Burgos-Vargas R, Shirazy K, et al. Assessment of clinical efficacy and safety in a randomized double-blind study of etanercept and sulfasalazine in patients with ankylosing spondylitis from Eastern/Central Europe, Latin America, and Asia. Rheumatol Int. 2016;36(5):643-51.

3. Calin A, Dijkmans BA, Emery P, Hakala M, Kalden J, Leirisalo-Repo M, et al. Outcomes of a multicentre randomised clinical trial of etanercept to treat ankylosing spondylitis. Ann Rheum Dis. 2004;63(12):1594-600.

4. Davis JC, Jr., Van Der Heijde D, Braun J, Dougados M, Cush J, Clegg DO, et al. Recombinant human tumor necrosis factor receptor (etanercept) for treating ankylosing spondylitis: a randomized, controlled trial. Arthritis Rheum. 2003;48(11):3230-6.

5. Dougados M, van der Heijde D, Sieper J, Braun J, Maksymowych WP, Citera G, et al. Symptomatic efficacy of etanercept and its effects on objective signs of inflammation in early nonradiographic axial spondyloarthritis: a multicenter, randomized, double-blind, placebo-controlled trial. Arthritis Rheumatol. 2014;66(8):2091-102.

6. Wei JC, Tsai WC, Citera G, Kotak S, Llamado L. Efficacy and safety of etanercept in patients from Latin America, Central Europe and Asia with early non-radiographic axial spondyloarthritis. Int J Rheum Dis. 2018;21(7):1443-51.

7. Song IH, Hermann K, Haibel H, Althoff CE, Listing J, Burmester G, et al. Effects of etanercept versus sulfasalazine in early axial spondyloarthritis on active inflammatory lesions as detected by whole-body MRI (ESTHER): a 48-week randomised controlled trial. Ann Rheum Dis. 2011;70(4):590-6.

8. Dougados M, Wood E, Combe B, Schaeverbeke T, Miceli-Richard C, Berenbaum F, et al. Evaluation of the nonsteroidal anti-inflammatory drug-sparing effect of etanercept in axial spondyloarthritis: results of the multicenter, randomized, double-blind, placebo-controlled SPARSE study. Arthritis Res Ther. 2014;16(6):481.

9. Dougados M, Braun J, Szanto S, Combe B, Elbaz M, Geher P, et al. Efficacy of etanercept on rheumatic signs and pulmonary function tests in advanced ankylosing spondylitis: results of a randomised double-blind placebo-controlled study (SPINE). Ann Rheum Dis. 2011;70(5):799-804.

10. van der Heijde D, Da Silva JC, Dougados M, Geher P, van der Horst-Bruinsma I, Juanola X, et al. Etanercept 50 mg once weekly is as effective as 25 mg twice weekly in patients with ankylosing spondylitis. Ann Rheum Dis. 2006;65(12):1572-7.

11. Sieper J, van der Heijde D, Dougados M, Mease PJ, Maksymowych WP, Brown MA, et al. Efficacy and safety of adalimumab in patients with non-radiographic axial spondyloarthritis: results of a randomised placebo-controlled trial (ABILITY-1). Ann Rheum Dis. 2013;72(6):815-22.

12. Landewé R, Sieper J, Mease P, Inman RD, Lambert RG, Deodhar A, et al. Efficacy and safety of continuing versus withdrawing adalimumab therapy in maintaining remission in patients with non-radiographic axial spondyloarthritis (ABILITY-3): a multicentre, randomised, double-blind study. Lancet. 2018;392(10142):134-44.

13. van der Heijde D, Kivitz A, Schiff MH, Sieper J, Dijkmans BA, Braun J, et al. Efficacy and safety of adalimumab in patients with ankylosing spondylitis: results of a multicenter, randomized, double-blind, placebo-controlled trial. Arthritis Rheum. 2006;54(7):2136-46.

14. Haibel H, Rudwaleit M, Listing J, Heldmann F, Wong RL, Kupper H, et al. Efficacy of adalimumab in the treatment of axial spondylarthritis without radiographically defined sacroiliitis: results of a twelve-week randomized, double-blind, placebo-controlled trial followed by an open-label extension up to week fifty-two. Arthritis Rheum. 2008;58(7):1981-91.

15. Horneff G, Fitter S, Foeldvari I, Minden K, Kuemmerle-Deschner J, Tzaribacev N, et al. Double-blind, placebo-controlled randomized trial with adalimumab for treatment of juvenile onset ankylosing spondylitis (JoAS): significant short term improvement. Arthritis Res Ther. 2012;14(5):R230.

16. Huang F, Gu J, Zhu P, Bao C, Xu J, Xu H, et al. Efficacy and safety of adalimumab in Chinese adults with active ankylosing spondylitis: results of a randomised, controlled trial. Ann Rheum Dis. 2014;73(3):587-94.

17. Deodhar A, Gensler LS, Kay J, Maksymowych WP, Haroon N, Landewé R, et al. A Fifty-Two-Week, Randomized, Placebo-Controlled Trial of Certolizumab Pegol in Nonradiographic Axial Spondyloarthritis. Arthritis Rheumatol. 2019;71(7):1101-11.

18. Landewé RB, van der Heijde D, Dougados M, Baraliakos X, Van den Bosch FE, Gaffney K, et al. Maintenance of clinical remission in early axial spondyloarthritis following certolizumab pegol dose reduction. Ann Rheum Dis. 2020;79(7):920-8.

19. Landewé R, Braun J, Deodhar A, Dougados M, Maksymowych WP, Mease PJ, et al. Efficacy of certolizumab pegol on signs and symptoms of axial spondyloarthritis including ankylosing spondylitis: 24-week results of a double-blind randomised placebo-controlled Phase 3 study. Ann Rheum Dis. 2014;73(1):39-47.

20. Bao C, Huang F, Khan MA, Fei K, Wu Z, Han C, et al. Safety and efficacy of golimumab in Chinese patients with active ankylosing spondylitis: 1-year results of a multicentre, randomized, double-blind, placebo-controlled phase III trial. Rheumatology (Oxford). 2014;53(9):1654-63.

21. Sieper J, van der Heijde D, Dougados M, Maksymowych WP, Scott BB, Boice JA, et al. A randomized, double-blind, placebo-controlled, sixteen-week study of subcutaneous golimumab in patients with active nonradiographic axial spondyloarthritis. Arthritis Rheumatol. 2015;67(10):2702-12.

22. Deodhar A, Reveille JD, Harrison DD, Kim L, Lo KH, Leu JH, et al. Safety and Efficacy of Golimumab Administered Intravenously in Adults with Ankylosing Spondylitis: Results through Week 28 of the GO-ALIVE Study. J Rheumatol. 2018;45(3):341-8.

23. Inman RD, Davis JC, Jr., Heijde D, Diekman L, Sieper J, Kim SI, et al. Efficacy and safety of golimumab in patients with ankylosing spondylitis: results of a randomized, double-blind, placebo-controlled, phase III trial. Arthritis Rheum. 2008;58(11):3402-12.

24. Tam LS, Shang Q, Kun EW, Lee KL, Yip ML, Li M, et al. The effects of golimumab on subclinical atherosclerosis and arterial stiffness in ankylosing spondylitis—a randomized, placebo-controlled pilot trial. Rheumatology (Oxford). 2014;53(6):1065-74.

25. van der Heijde D, Dijkmans B, Geusens P, Sieper J, DeWoody K, Williamson P, et al. Efficacy and safety of infliximab in patients with ankylosing spondylitis: results of a randomized, placebo-controlled trial (ASSERT). Arthritis Rheum. 2005;52(2):582-91.

26. Burgos-Vargas R, Loyola-Sanchez A, Ramiro S, Reding-Bernal A, Alvarez-Hernandez E, van der Heijde D, et al. A randomized, double-blind, placebo-controlled 12-week trial of infliximab in patients with juvenile-onset spondyloarthritis. Arthritis Res Ther. 2022;24(1):187.

27. Giardina AR, Ferrante A, Ciccia F, Impastato R, Miceli MC, Principato A, et al. A 2-year comparative open label randomized study of efficacy and safety of etanercept and infliximab in patients with ankylosing spondylitis. Rheumatol Int. 2010;30(11):1437-40.

28. Sieper J, Lenaerts J, Wollenhaupt J, Rudwaleit M, Mazurov VI, Myasoutova L, et al. Efficacy and safety of infliximab plus naproxen versus naproxen alone in patients with early, active axial spondyloarthritis: results from the double-blind, placebo-controlled INFAST study, Part 1. Ann Rheum Dis. 2014;73(1):101-7.

29. Inman RD, Maksymowych WP. A double-blind, placebo-controlled trial of low dose infliximab in ankylosing spondylitis. J Rheumatol. 2010;37(6):1203-10.

30. Marzo-Ortega H, McGonagle D, Jarrett S, Haugeberg G, Hensor E, O'Connor P, et al. Infliximab in combination with methotrexate in active ankylosing spondylitis: a clinical and imaging study. Ann Rheum Dis. 2005;64(11):1568-75.

31. van der Heijde D, Cheng-Chung Wei J, Dougados M, Mease P, Deodhar A, Maksymowych WP, et al. Ixekizumab, an interleukin-17A antagonist in the treatment of ankylosing spondylitis or radiographic axial spondyloarthritis in patients previously untreated with biological disease-modifying anti-rheumatic drugs (COAST-V): 16 week results of a phase 3 randomised, double-blind, active-controlled and placebo-controlled trial. Lancet. 2018;392(10163):2441-51.

32. Deodhar A, Poddubnyy D, Pacheco-Tena C, Salvarani C, Lespessailles E, Rahman P, et al. Efficacy and Safety of Ixekizumab in the Treatment of Radiographic Axial Spondyloarthritis: Sixteen-Week Results From a Phase III Randomized, Double-Blind, Placebo-Controlled Trial in Patients With Prior Inadequate Response to or Intolerance of Tumor Necrosis Factor Inhibitors. Arthritis Rheumatol. 2019;71(4):599-611.

33. Deodhar A, van der Heijde D, Gensler LS, Kim TH, Maksymowych WP, Østergaard M, et al. Ixekizumab for patients with non-radiographic axial spondyloarthritis (COAST-X): a randomised, placebo-controlled trial. Lancet. 2020;395(10217):53-64.

34. Erdes S, Nasonov E, Kunder E, Pristrom A, Soroka N, Shesternya P, et al. Primary efficacy of netakimab, a novel interleukin-17 inhibitor, in the treatment of active ankylosing spondylitis in adults. Clin Exp Rheumatol. 2020;38(1):27-34.

35. Baeten D, Baraliakos X, Braun J, Sieper J, Emery P, van der Heijde D, et al. Anti-interleukin-17A monoclonal antibody secukinumab in treatment of ankylosing spondylitis: a randomised, double-blind, placebo-controlled trial. Lancet. 2013;382(9906):1705-13.

36. Baeten D, Sieper J, Braun J, Baraliakos X, Dougados M, Emery P, et al. Secukinumab, an Interleukin-17A Inhibitor, in Ankylosing Spondylitis. N Engl J Med. 2015;373(26):2534-48.

37. Pavelka K, Kivitz A, Dokoupilova E, Blanco R, Maradiaga M, Tahir H, et al. Efficacy, safety, and tolerability of secukinumab in patients with active ankylosing spondylitis: a randomized, double-blind phase 3 study, MEASURE 3. Arthritis Res Ther. 2017;19(1):285.

38. Kivitz AJ, Wagner U, Dokoupilova E, Supronik J, Martin R, Talloczy Z, et al. Efficacy and Safety of Secukinumab 150 mg with and Without Loading Regimen in Ankylosing Spondylitis: 104-week Results from MEASURE 4 Study. Rheumatol Ther. 2018;5(2):447-62.

39. Huang F, Sun F, Wan WG, Wu LJ, Dong LL, Zhang X, et al. Secukinumab provided significant and sustained improvement in the signs and symptoms of ankylosing spondylitis: results from the 52-week, Phase III China-centric study, MEASURE 5. Chin Med J (Engl). 2020;133(21):2521-31.

40. Deodhar A, Blanco R, Dokoupilová E, Hall S, Kameda H, Kivitz AJ, et al. Improvement of Signs and Symptoms of Nonradiographic Axial Spondyloarthritis in Patients Treated With Secukinumab: Primary Results of a Randomized, Placebo-Controlled Phase III Study. Arthritis Rheumatol. 2021;73(1):110-20.

41. van der Heijde D, Gensler LS, Deodhar A, Baraliakos X, Poddubnyy D, Kivitz A, et al. Dual neutralisation of interleukin-17A and interleukin-17F with bimekizumab in patients with active ankylosing spondylitis: results from a 48-week phase IIb, randomised, double-blind, placebo-controlled, dose-ranging study. Ann Rheum Dis. 2020;79(5):595-604.

42. Wei JC, Kim TH, Kishimoto M, Ogusu N, Jeong H, Kobayashi S. Efficacy and safety of brodalumab, an anti-IL17RA monoclonal antibody, in patients with axial spondyloarthritis: 16-week results from a randomised, placebo-controlled, phase 3 trial. Ann Rheum Dis. 2021;80(8):1014-21.

43. Deodhar A, Sliwinska-Stanczyk P, Xu H, Baraliakos X, Gensler LS, Fleishaker D, et al. Tofacitinib for the treatment of ankylosing spondylitis: a phase III, randomised, double-blind, placebo-controlled study. Ann Rheum Dis. 2021;80(8):1004-13.

44. van der Heijde D, Deodhar A, Wei JC, Drescher E, Fleishaker D, Hendrikx T, et al. Tofacitinib in patients with ankylosing spondylitis: a phase II, 16-week, randomised, placebo-controlled, dose-ranging study. Ann Rheum Dis. 2017;76(8):1340-7.

45. van der Heijde D, Baraliakos X, Gensler LS, Maksymowych WP, Tseluyko V, Nadashkevich O, et al. Efficacy and safety of filgotinib, a selective Janus kinase 1 inhibitor, in patients with active ankylosing spondylitis (TORTUGA): results from a randomised, placebo-controlled, phase 2 trial. Lancet. 2018;392(10162):2378-87.

46. van der Heijde D, Song IH, Pangan AL, Deodhar A, van den Bosch F, Maksymowych WP, et al. Efficacy and safety of upadacitinib in patients with active ankylosing spondylitis (SELECT-AXIS 1): a multicentre, randomised, double-blind, placebo-controlled, phase 2/3 trial. Lancet. 2019;394(10214):2108-17.

47. van der Heijde D, Baraliakos X, Sieper J, Deodhar A, Inman RD, Kameda H, et al. Efficacy and safety of upadacitinib for active ankylosing spondylitis refractory to biological therapy: a double-blind, randomised, placebo-controlled phase 3 trial. Ann Rheum Dis. 2022;81(11):1515-23.

48. Deodhar A, Van den Bosch F, Poddubnyy D, Maksymowych WP, van der Heijde D, Kim TH, et al. Upadacitinib for the treatment of active non-radiographic axial spondyloarthritis (SELECT-AXIS 2): a randomised, double-blind, placebo-controlled, phase 3 trial. Lancet. 2022;400(10349):369-79.

49. Sieper J, Porter-Brown B, Thompson L, Harari O, Dougados M. Assessment of short-term symptomatic efficacy of tocilizumab in ankylosing spondylitis: results of randomised, placebo-controlled trials. Ann Rheum Dis. 2014;73(1):95-100.

50. Baeten D, Østergaard M, Wei JC, Sieper J, Järvinen P, Tam LS, et al. Risankizumab, an IL-23 inhibitor, for ankylosing spondylitis: results of a randomised, double-blind, placebo-controlled, proof-of-concept, dose-finding phase 2 study. Ann Rheum Dis. 2018;77(9):1295-302.

51. Deodhar A, Gensler LS, Sieper J, Clark M, Calderon C, Wang Y, et al. Three Multicenter, Randomized, Double-Blind, Placebo-Controlled Studies Evaluating the Efficacy and Safety of Ustekinumab in Axial Spondyloarthritis. Arthritis Rheumatol. 2019;71(2):258-70.

52. Pathan E, Abraham S, Van Rossen E, Withrington R, Keat A, Charles PJ, et al. Efficacy and safety of apremilast, an oral phosphodiesterase 4 inhibitor, in ankylosing spondylitis. Ann Rheum Dis. 2013;72(9):1475-80.

53. Taylor PC, van der Heijde D, Landewé R, McCue S, Cheng S, Boonen A. A Phase III Randomized Study of Apremilast, an Oral Phosphodiesterase 4 Inhibitor, for Active Ankylosing Spondylitis. J Rheumatol. 2021;48(8):1259-67.

54. Khanna Sharma S, Kadiyala V, Naidu G, Dhir V. A randomized controlled trial to study the efficacy of sulfasalazine for axial disease in ankylosing spondylitis. Int J Rheum Dis. 2018;21(1):308-14.

55. IntHout J, Ioannidis JPA, Rovers MM, Goeman JJ. Plea for routinely presenting prediction intervals in meta-analysis. BMJ Open. 2016;6(7):e010247.

56. Lin L. Use of Prediction Intervals in Network Meta-analysis. JAMA Netw Open. 2019;2(8):e199735.

57. Salanti G, Ades AE, Ioannidis JPA. Graphical methods and numerical summaries for presenting results from multiple-treatment meta-analysis: an overview and tutorial. Journal of Clinical Epidemiology. 2011;64(2):163-71.
